# NDFIP1 limits cellular TAZ accumulation via exosomal sorting to inhibit NSCLC proliferation

**DOI:** 10.1093/procel/pwac017

**Published:** 2022-09-21

**Authors:** Yirui Cheng, Xin Lu, Fan Li, Zhuo Chen, Yanshuang Zhang, Qing Han, Qingyu Zeng, Tingyu Wu, Ziming Li, Shun Lu, Cecilia Williams, Weiliang Xia

**Affiliations:** State Key Laboratory of Oncogenes and Related Genes, Ren Ji Hospital, School of Medicine and School of Biomedical Engineering, Shanghai Jiao Tong University, Shanghai 200030, China; State Key Laboratory of Oncogenes and Related Genes, Ren Ji Hospital, School of Medicine and School of Biomedical Engineering, Shanghai Jiao Tong University, Shanghai 200030, China; State Key Laboratory of Oncogenes and Related Genes, Ren Ji Hospital, School of Medicine and School of Biomedical Engineering, Shanghai Jiao Tong University, Shanghai 200030, China; State Key Laboratory of Oncogenes and Related Genes, Ren Ji Hospital, School of Medicine and School of Biomedical Engineering, Shanghai Jiao Tong University, Shanghai 200030, China; State Key Laboratory of Oncogenes and Related Genes, Ren Ji Hospital, School of Medicine and School of Biomedical Engineering, Shanghai Jiao Tong University, Shanghai 200030, China; State Key Laboratory of Oncogenes and Related Genes, Ren Ji Hospital, School of Medicine and School of Biomedical Engineering, Shanghai Jiao Tong University, Shanghai 200030, China; Shanghai Skin Disease Hospital, Tongji University School of Medicine, Shanghai 200092, China; State Key Laboratory of Oncogenes and Related Genes, Ren Ji Hospital, School of Medicine and School of Biomedical Engineering, Shanghai Jiao Tong University, Shanghai 200030, China; Shanghai Lung Cancer Center, Shanghai Chest Hospital, Shanghai Jiao Tong University, Shanghai 200030, China; Shanghai Lung Cancer Center, Shanghai Chest Hospital, Shanghai Jiao Tong University, Shanghai 200030, China; Department of Protein Science, KTH Royal Institute of Technology, Science for Life Laboratory, Solna 170 70, Sweden; State Key Laboratory of Oncogenes and Related Genes, Ren Ji Hospital, School of Medicine and School of Biomedical Engineering, Shanghai Jiao Tong University, Shanghai 200030, China

**Keywords:** NDFIP1, TAZ, NSCLC, exosome, cargo sorting

## Abstract

NDFIP1 has been previously reported as a tumor suppressor in multiple solid tumors, but the function of NDFIP1 in NSCLC and the underlying mechanism are still unknown. Besides, the WW domain containing proteins can be recognized by NDFIP1, resulted in the loading of the target proteins into exosomes. However, whether WW domain-containing transcription regulator 1 (WWTR1, also known as TAZ) can be packaged into exosomes by NDFIP1 and if so, whether the release of this oncogenic protein via exosomes has an effect on tumor development has not been investigated to any extent. Here, we first found that *NDFIP1* was low expressed in NSCLC samples and cell lines, which is associated with shorter OS. Then, we confirmed the interaction between TAZ and NDFIP1, and the existence of TAZ in exosomes, which requires NDFIP1. Critically, knockout of *NDFIP1* led to TAZ accumulation with no change in its mRNA level and degradation rate. And the cellular TAZ level could be altered by exosome secretion. Furthermore, NDFIP1 inhibited proliferation *in vitro* and *in vivo*, and silencing *TAZ* eliminated the increase of proliferation caused by *NDFIP1* knockout. Moreover, TAZ was negatively correlated with NDFIP1 in subcutaneous xenograft model and clinical samples, and the serum exosomal TAZ level was lower in NSCLC patients. In summary, our data uncover a new tumor suppressor, NDFIP1 in NSCLC, and a new exosome-related regulatory mechanism of TAZ.

## Introduction

Lung cancer occurrence and mortality ranks top in China and around the world (Sung et al., 2021). About 85% of lung cancers are non-small cell lung cancer (NSCLC), which can be subdivided into adenocarcinoma (LADC), squamous cell carcinoma (LSQ), and large cell carcinoma (LLC) (Lewis et al., 2014). Over the past 20 years, as the treatment paradigm of lung cancer shifted from cytotoxic therapy to personalized targeted therapies, the curative effect has been substantially progressed (Herbst et al., 2018). Yet, only certain populations of patients with specific druggable genomic alterations are benefited. Therefore, the identification of new therapeutic targets and its molecular mechanisms remains increasingly needed to expand the treatable population.

The Hippo signaling pathway controls growth and organ size, thus its dysregulation often leads to cell overgrowth and tumorigenesis (Chen et al., 2019). WW domain-containing proteins, Yes-associated protein (YAP), and Transcriptional coactivator with PDZ-binding motif (TAZ) are core components of the Hippo pathway. In general, when the pathway is turned off, a cascade of kinases (MST1/2 and LATS1/2) are dephosphorylated, followed by nuclear translocation of the dephosphorylated transcriptional coactivators YAP/TAZ, where they transcriptionally activate tumor promoting genes or repress tumor suppressive genes Zanconato et al., 2016; Xie et al., 2018). In solid cancers, especially in NSCLC, YAP/TAZ are not only upregulated by Hippo signaling, but also by other molecular mechanisms (Lu et al., 2018; Ma et al., 2020; Pocaterra et al., 2020). Interestingly, increased level of tumor YAP and/or TAZ is hardly caused by genomic amplification (Lo Sardo et al., 2018), thus, the regulatory mechanisms of YAP/TAZ are complicated but important in solid tumor, such as NSCLC.

Nedd4 family-interacting protein 1 (NDFIP1) is an adaptor of E3 ubiquitin ligases Nedd4 family, linking Nedd4 with target proteins to facilitate the ubiquitination (Harvey et al., 2002). As previously demonstrated, NDFIP1 participates in inflammatory, neurological, and autoimmune diseases (Howitt et al., 2009; Oliver et al., 2006; Wagle et al., 2018), but recently, the downregulation of *NDFIP1* promotes proliferation, invasion, epithelial mesenchymal transition (EMT), or glycolysis in several forms of cancer (Peng et al., 2017; Zhang et al., 2019; Ben et al., 2020). However, the function of NDFIP1 in NSCLC has not been investigated to any extent.

In previous reports, NDFIP1 is identified to function in the loading of proteins into exosomes through the binding of WW domain in target proteins to PPxY motif in NDFIP1 (Putz et al., 2008, 2012; Sterzenbach et al., 2017). Given that YAP and TAZ are classic WW domain-containing proteins (Salah et al., 2012), it is reasonable to hypothesize that YAP/TAZ can be recognized by NDFIP1 and subsequently loaded into exosomes. And if so, considering the oncogenic function of YAP/TAZ in NSCLC, why tumor cells expel this oncoprotein needs to be further explored.

Here, we first verified the low expression of *NDFIP1* in NSCLC, the binding of NDFIP1 with TAZ and the recruitment of TAZ, but little YAP into NSCLC exosomes. Then combining evidences from clinical data, *in vitro* and *in vivo* experiments, we revealed that lower NDFIP1 led to lower TAZ packaged in exosomes and higher TAZ accumulation in cells, promoting cell proliferation and tumor growth eventually. Taken together, our data have disclosed NDFIP1 as a novel tumor suppressor and proposed a new exosome-related regulatory mechanism of TAZ, providing new insights for the development of biomarkers and treatment strategies of NSCLC.

## Results

### NDFIP1 is downregulated in NSCLC, which is associated with shorter OS

**Figure 1.**
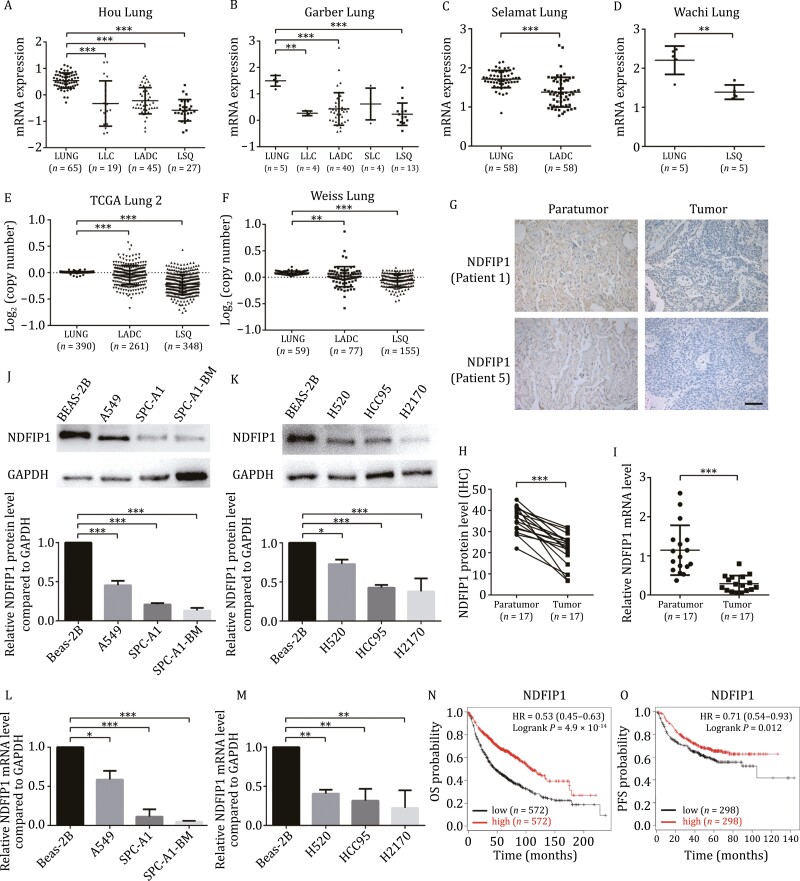
**NDFIP1 is low expressed in human NSCLC samples and cell lines, and lower NDFIP1 is associated with shorter OS.** (A–F) Expression of NDFIP1 and copy number analyses of NDFIP1 in Oncomine databases with provisional LSQ, LADC, LLC, and SLC cohorts. Expression (A) in Hou Lung datasets, (B) in Garber Lung datasets, (C) in Selamat Lung datasets, (D) in Wachi Lung datasets, copy number (E) in TCGA Lung 2 datasets, (F) in Weiss Lung datasets. Bars represent the mean ± SD. **P < 0.01. ***P < 0.001. (G) IHC of NDFIP1 in tumor and paratumor tissues from patients 1 and 5. Scale bars: 200 μm. (H) Quantification of IHC staining intensity for NDFIP1 in 17 paired NSCLC tissues. ***P < 0.001. (I) *NDFIP1* mRNA level in NSCLC tissues and matched non-tumor tissues (*n* = 17). Bars represent the mean ± SD. ***P < 0.001. (J–M) NDFIP1 (J) protein and (L) mRNA level in (K) LADC cell lines, A549, SPC-A1, SPC-A1-BM, (M) LSQ cell lines, H520, HCC95, H2170 and bronchial epithelial cell line Beas-2B. Bars represent the mean ± SD (*n* = 3). *P < 0.01. **P < 0.01. ***P < 0.001. (N–O) Kaplan–Meier analysis of NDFIP1 level on the (N) overall survival (OS) and (O) progression-free survival (PFS) in lung cancer patients.

As previously reported, NDFIP1 is regarded as a tumor suppressor in uveal melanoma, hepatocellular carcinoma, pancreatic ductal adenocarcinoma, and breast cancer (Peng et al., 2017; Zhang et al., 2019; Ben et al., 2020; Tian et al., 2020). To explore the role of NDFIP1 in NSCLC, we first analyzed the *NDFIP1* mRNA expression and *NDFIP1* DNA copy number in the different datasets of Oncomine. Decreased *NDFIP1* expression levels were found in NSCLC in Hou Lung, Garber Lung, Selamat Lung, and Wachi Lung datasets (Fig. 1A–D). Consistently, *NDFIP1* copy number was also decreased in TCGA Lung 2 and Weiss Lung datasets (Fig. 1E and 1F). Moreover, we examined the NDFIP1 levels in 17 pairs of samples from NSCLC patients. NDFIP1 protein levels were lower in tumor samples than that in matched paratumor samples (Figs. 1G, 1H, and S1). And tumor *NDFIP1* mRNA levels were lower in tumor tissues (Fig. 1I). Similarly, in NSCLC cells, the NDFIP1 protein levels and *NDFIP1* mRNA levels were both decreased in LADC (A549, SPC-A1, SPC-A1-BM) and LSQ (H520, HCC95, H2170) cell lines compared with the normal bronchial epithelial cell line, Beas-2B (Fig. 1J–M). Hypoxia also reduced the NDFIP1 protein level and *NDFIP1* mRNA level in SPC-A1-BM cells (Fig. S2A and S2B). Furthermore, the survival curves from the Kaplan–Meier plotter revealed that higher *NDFIP1* level (top 50%) was associated with better overall survival (OS; *n* = 1,144) and progression-free survival (PFS; *n* = 596) (Fig. 1N and 1O)). Overall, these findings suggested NDFIP1 as a tumor suppressor in NSCLC.

### NDFIP1 interacts with TAZ and is responsible for the recruitment of TAZ into exosomes

**Figure 2.**
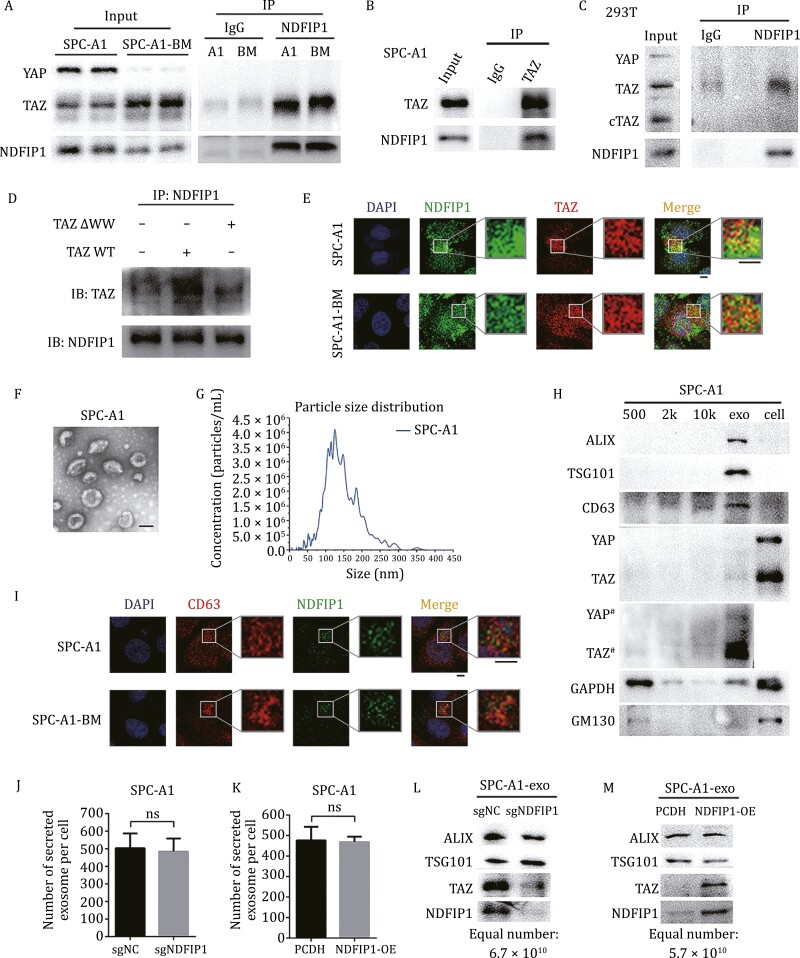
**NDFIP1 interacts with TAZ, and TAZ can be sorted into exosomes, which requires NDFIP1.** (A) The binding of NDFIP1 to TAZ in SPC-A1 and SPC-A1-BM. (B) The converse binding of TAZ to NDFIP1 in SPC-A1. (C) The binding of NDFIP1 to TAZ in 293T. (D) The binding of NDFIP1 to TAZ WT, but not to the TAZ ΔWW in SPC-A1. (E) IF co-localization of NDFIP1 and TAZ in SPC-A1 and SPC-A1-BM. Scale bars: 5 μm. (F) TEM image of SPC-A1 exosomes. Scale bars: 100 nm. (G) The particle size distribution of exosomes from SPC-A1. (H) The protein levels of YAP, TAZ, exosome-specific proteins, ALIX, TSG101, CD63, a cell internal reference, GAPDH and a Golgi marker, GM130 in different components of SPC-A1, including the pellets of centrifugation at 500 ×*g*, 2,000 ×*g*, 10,000 ×*g*, exosomes and cells. # represents longer exposure. (I) IF co-localization of CD63 and TAZ in SPC-A1 and SPC-A1-BM. Scale bars: 5 μm. (J–K) The average number of secreted exosomes per cell of (J) the control (sgNC) and *NDFIP1* knockout (sgNDFIP1) and (K) the control (PCDH) and *NDFIP1* overexpression (NDFIP1-OE) SPC-A1 cells. Bars represent the mean ± SD (*n* = 3). (J) ns, *P* = 0.1977. (K) ns, *P* = 0.8157. (L–M) The TAZ and NDFIP1 protein levels in equal number of exosomes from the (L) control (sgNC) and *NDFIP1* knockout (sgNDFIP1) SPC-A1 cells, and (M) from the control (PCDH) and *NDFIP1* overexpression (NDFIP1-OE) SPC-A1 cells.

Given that NDFIP1 contains PPxY motifs (Harvey et al., 2002), and YAP/TAZ are classic WW-domain containing proteins that could be recognized by PPxY motifs (Salah et al., 2012), co-IP assays were performed in SPC-A1, SPC-A1-BM, and A549 to explore if NDFIP1 could interact with YAP/TAZ. The results verified the binding of NDFIP1 to TAZ, but not YAP (Figs. 2A and S3A). Furthermore, the binding of TAZ to NDFIP1 reversely was confirmed (Figs. 2B and S3B). Interestingly, NDFIP1 interacted with TAZ, rather than cTAZ (C-terminus of TAZ) (Fig. 2C), a short variant of TAZ lacking an intact WW domain (Fang et al., 2019), indicating the recognition of TAZ by NDFIP1 requires the WW domain. Moreover, the TAZ WT (wide type) and TAZ ΔWW (TAZ mutation with WW-domain deletion) plasmids (Varelas et al., 2008) were transfected in SPC-A1 cells (Fig. S4), and the co-IP results showed that NDFIP1 interacted with TAZ WT, but not with the TAZ ΔWW (Fig. 2D, which confirmed that NDFIP1 recognizes TAZ through its WW-domain. Then, the subcellular co-localization of NDFIP1 and TAZ also provided additional evidence supporting the binding of NDFIP1 with TAZ (Fig. 2E).

Next, to verify if TAZ can be found in exosomes, exosomes from conditioned media of different lung cancer cells (SPC-A1, A549, SPC-A1-BM, H1581, and H520) were isolated by ultracentrifugation method (Fig. S3A) and characterized by TEM, NTA and Western blot (Figs. 2E–G and S5B–G). Exosomes displayed the classical “cup-shaped” morphology (Figs. 2F and S5B) and the size of exosomes mostly distributed between 30 and 200 nm (Figs. 2G and S5C). Furthermore, it was found that TAZ, but little YAP, were mostly enriched in exosome pellets rather than other fractions (Figs. 2H and S5D–G), such as intact cells in suspension during culture (500 ×*g*), cell debris (2,000 ×*g*), and extracellular vesicles (10,000 ×*g*) (Menck et al., 2017). Meanwhile, exosome-specific proteins, ALIX, TSG101, CD63, CD9, and CD81, were also concentrated in exosome pellets whereas GAPDH (a cell internal reference) and GM130 (a Golgi marker) (Putz et al., 2012) levels were much lower in exosomes than in other components (Figs. 2H and S5D–G). In addition, the co-localization of TAZ and CD63 in SPC-A1 and SPC-A-1-BM cells reconfirmed the presence of TAZ in exosomes (Fig. 2I). Furthermore, the knockout and overexpression of *NDFIP1* in SPC-A1 cells, respectively, resulted in the decrease and increase of TAZ in equal number of exosomes (Fig. 2L and 2M), but have no significant effect on the exosomes number secreted by single cell (Fig. 2J and 2K). Similarly, in A549, the knockdown and overexpression of *NDFIP1* did not affect the number of exosomes secreted per cell (Fig. S6A and S6B), but, respectively, inhibited and promoted the export of TAZ into exosomes (Fig. S6C and S6D). Also, the TAZ level in equal number of exosomes was lower in tumor cells with lower *NDFIP1* expression (Figs. 1J, S2A, S7A, and S7B). Thus, these observations indicated that NDFIP1 is responsible for exosome-mediated release of TAZ in NSCLC cells.

### NDFIP1 determines the cellular accumulation of TAZ through exosomes

**Figure 3.**
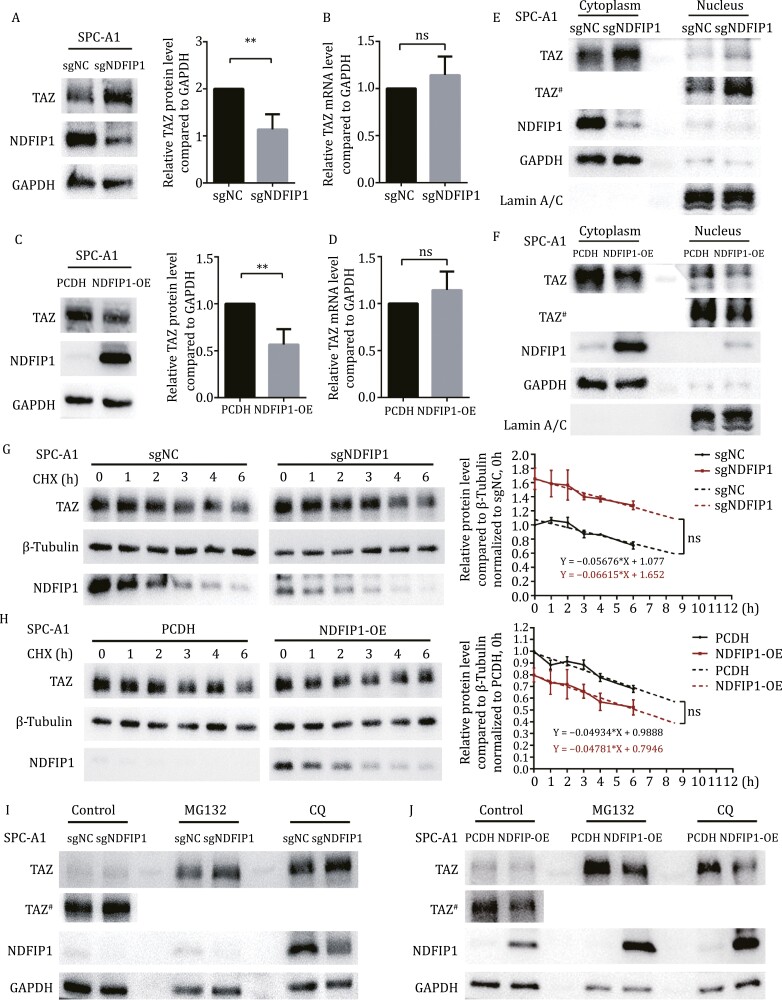
**NDFIP1 negatively regulates cellular TAZ, which is irrelevant to transcriptional level and degradation rate of TAZ.** (A and C) Protein levels of TAZ and NDFIP1 in (A) control (sgNC) and *NDFIP1* knockout (sgNDFIP1) SPC-A1 cells, (C) control (PCDH) and *NDFIP1* overexpression (NDFIP1-OE) SPC-A1 cells. The relative quantification of TAZ is shown on the right. Bars represent the mean ± SD (*n* = 3). **P < 0.01. (B and D) Relative mRNA levels of *TAZ* in these four groups. Bars represent the mean ± SD (*n* = 3). (B) ns, *P* = 0.8114. (D) ns, *P* = 0.2795. (E–F) Protein levels of TAZ and NDFIP1 in the cytoplasm and the nucleus fraction in these four groups. ^#^ represents longer exposure. (G–H) CHX chase experiment on TAZ in these four groups. The relative quantification of TAZ was plotted on the right. Bars represent the mean ± SD (*n* = 3). (G) ns *P* = 0.5752. (H) ns *P* = 0.8843. (I–J) Protein levels of TAZ and NDFIP1 in these four groups with or without a proteasome activity inhibitor, MG132 and a lysosome activity inhibitor, CQ. # represents longer exposure.

We next examined the influence of NDFIP1 on cellular TAZ. The knockout of *NDFIP1* in SPC-A1 cells and the knockdown of *NDFIP1* in A549 cells resulted in the increase of TAZ protein level, rather than *TAZ* mRNA level (Figs. 3A, 3B, S8A, and S8B). And the overexpression of *NDFIP1* in SPC-A1 cells and A549 cells caused the reduction at protein levels of TAZ with no significant alterations in the mRNA level (Figs. 3C, 3D, S3C, and S3D). Therefore, in contrast to exosomal TAZ, NDFIP1 limited cellular TAZ accumulation, and the *NDFIP1*-induced TAZ protein differences cannot be explained by the regulation at transcriptional level.

Moreover, considering TAZ as a transcription factor that needs to translocate into the nucleus to function, we then extracted the nuclear and cytoplasmic fractions separately, with GAPDH and Lamin A/C as cytoplasmic and nuclear loading control, respectively (Zhang et al., 2018). And it was found that the increase and decrease of TAZ induced by *NDFIP1* not only occurred in the cytoplasm but also in the nucleus (Figs. 3E, 3F, S8E, and S8F).

Next, given that NDFIP1 mediates the degradation of certain proteins (O’Leary et al., 2016; Gorla et al., 2019), we evaluated potential differences in TAZ protein degradation. Cycloheximide [CHX, an inhibitor of protein synthesis (Liu et al., 2019)] chase experiments revealed that the degradation rate of TAZ was hardly affected by NDFIP1 (Fig. 3G and 3H). In addition, the two main pathways for protein degradation are the proteasome and the lysosome (Ciechanover, 2005). However, the differences in the TAZ protein level caused by *NDFIP1* were unaltered whether the MG132, a proteasome activity inhibitor (Lee and Goldberg, 1998), and Chloroquine (CQ), a lysosome activity inhibitor (Banik et al., 2020), existed or not (Fig. 3I and 3J). Together, the regulation of the cellular TAZ protein level was unlikely through its degradation.

Furthermore, the decrease in TAZ induced by *NDFIP1* overexpression in SPC-A1 and A549 cells were increased by GW4869, an exosome secretion inhibitor (Fig. S9A–C) (Trajkovic et al., 2008). And the knockdown of *RAB27B*, an important molecule to control exosome secretion (Ostrowski et al., 2010), also retained more TAZ intracellularly in the *NDFIP1* overexpressed SPC-A1 and A549 cells (Fig. S9D–F). Taken together, cellular TAZ accumulation could be negatively regulated by exosome secretion. Thus, the differences in the TAZ level in exosomes appear to underlie the *NDFIP1*-mediated cellular TAZ levels to be either up- or downregulated.

### NDFIP1 inhibits the proliferation of NSCLC cells *in vitro* and *in vivo*

**Figure 4.**
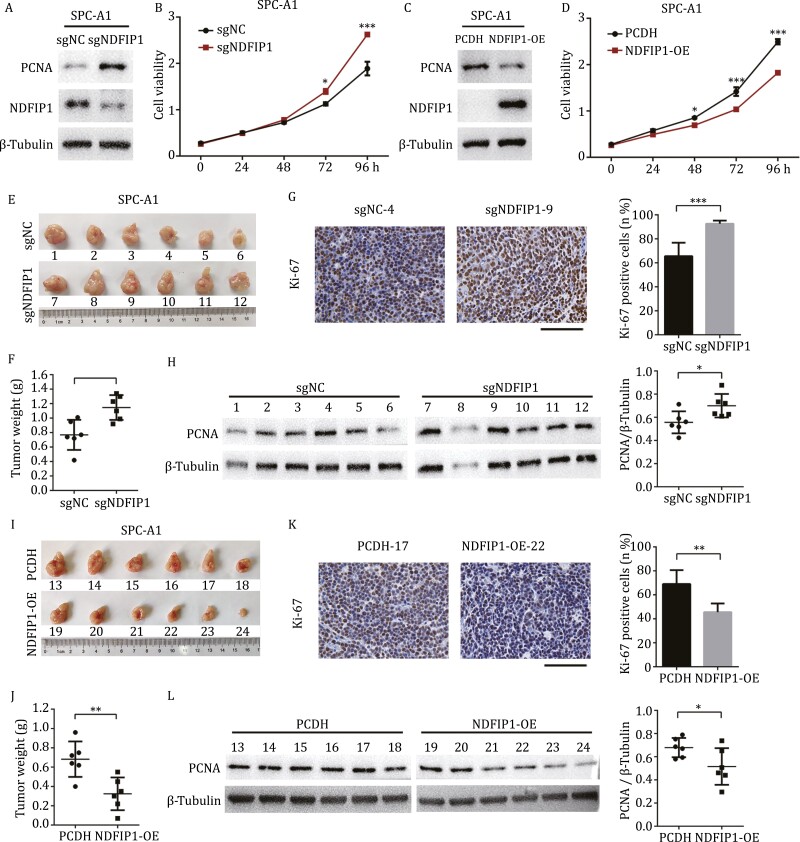
**Downregulation of NDFIP1 promotes cell proliferation *in vitro* and *in vivo*.** (A and C) Protein levels of a proliferation marker, PCNA in (A) control (sgNC) and *NDFIP1* knockout (sgNDFIP1) SPC-A1 cells, (C) control (PCDH) and *NDFIP1* overexpression (NDFIP1-OE) SPC-A1 cells. (B and D) Cell viability assay of these four groups. Bars represent the mean ± SD (*n* = 3). *P < 0.05. ***P < 0.001. (E, I), the images of tumors from subcutaneous mouse xenograft model of (E) control (sgNC) and *NDFIP1* knockout (sgNDFIP1) SPC-A1 cells, (I) control (PCDH) and *NDFIP1* overexpression (NDFIP1-OE) SPC-A1 cells. (F, J) the weight of these tumors. Bars represent the mean ± SD (*n* = 6). **P < 0.01. (G and K) Representative IHC images of tumors from these four groups. Scale bars: 100 μm. The quantification of Ki-67-positive cells is plotted on the right. Bars represent the mean ± SD (*n* = 6). **P < 0.01. ***P < 0.001. (H and L) The PCNA protein level in these tumors. The relative quantification of PCNA is plotted on the right. Bars represent the mean ± SD (*n* = 6). *P < 0.05.

Since TAZ mainly drives tumor growth, we further tested whether NDFIP1 had an effect on NSCLC proliferation. The knockout of *NDFIP1* in SPC-A1 cells led to a higher level of PCNA, a proliferation marker, along with a significant increase in cell viability (Fig. 4A and 4B), while overexpressing *NDFIP1* caused the opposite outcome (Fig. 4C and 4D). Similarly, in A549 cells, the increased PCNA level and cell viability occurred in *NDFIP1* knockdown cells (Fig. S10A and S10B), and *NDFIP1* overexpression reduced the level of PCNA and inhibited the cell viability (Fig. S10C and S10D).

Moreover, silencing *TAZ* eliminated the increase of proliferation caused by *NDFIP1* knockout in SPC-A1 cells (Fig. S11A and S11B) and *NDFIP1* knockdown in A549 cells (Fig. S11C and S11D). So, the NDFIP1-mediated cell proliferation could be inhibited by TAZ reduction.

Then the stably transfected SPC-A1 cells were subcutaneously injected into the right flanks of BALB/c nude mice (*n* = 6) (Fig. S12), and a marked increase in tumor size and weight was observed in mice receiving the *NDFIP1* knockout cells (Fig. 4E and 4F). Not only that, the PCNA expressions were also increased, accompanied by higher levels of Ki-67 in the mice implanted with *NDFIP1* knockout cells (Figs. 4G, 4H and S13A). And vice versa, the overexpression of *NDFIP1* resulted in smaller tumor size and weight, lower level of PCNA and Ki-67 *in vivo* (Figs. 4I–L and S13B).

### NDFIP1 switches the abundances of intra- and extra-cellular TAZ *in vivo*

**Figure 5.**
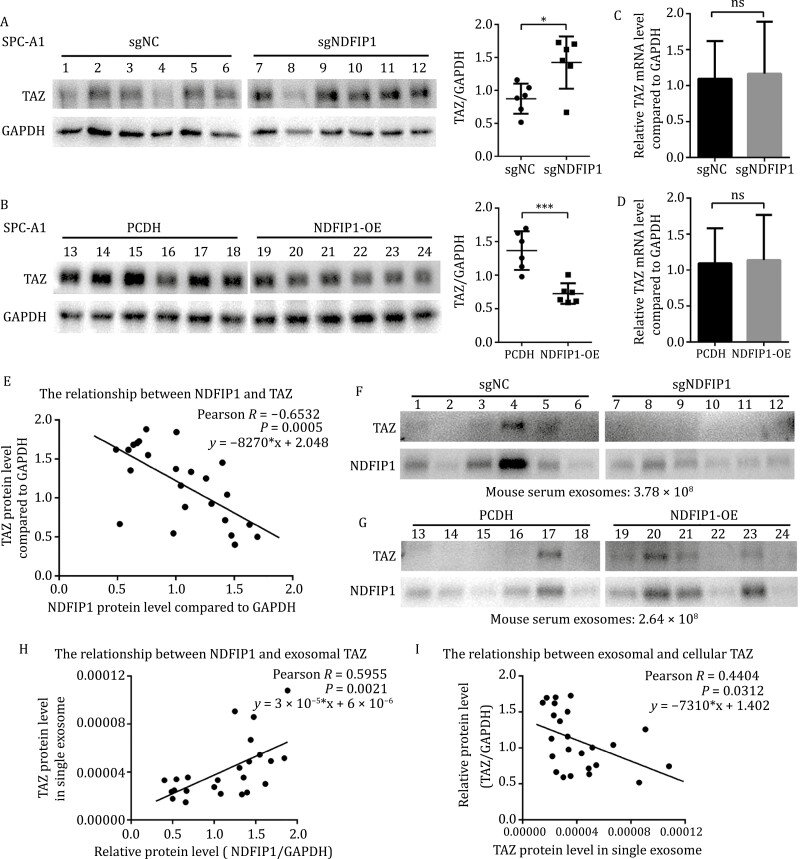
**NDFIP1 acts as a mediator of intra- and extracellular TAZ *in vivo*.** (A–B) The TAZ protein level in subcutaneous tumors from mice receiving (A) control (sgNC) and *NDFIP1* knockout (sgNDFIP1) SPC-A1 cells, (C) control (PCDH) and *NDFIP1* overexpression (NDFIP1-OE) SPC-A1 cells. The relative quantification of TAZ was plotted on the right. Bars represent the mean ± SD (*n* = 6). *P < 0.05. ***P < 0.001. (C–D) The TAZ mRNA level in these tumors. Bars represent the mean ± SD (*n* = 6). (C) ns, *P* = 0.8524. (D) ns, *P* = 0.8891. (E) Linear correlation analysis of TAZ with NDFIP1 in these tumor cells (*n* = 24). (F–G) The TAZ protein level in equal number of mouse serum exosomes from these four groups. (H–I) Linear correlation analysis of (H) TAZ level in single exosome from mouse serum with cellular NDFIP1 level (*n* = 24). (I) TAZ in these tumor cells with TAZ in single exosome from matched mouse serum (*n* = 24).

Now that *in vitro* evidences have indicated NDFIP1 mediates TAZ packaging into exosomes and accumulation in cells, we next test this mechanism *in vivo*. Expectedly, the protein levels of TAZ were generally upregulated in the *NDFIP1* knockout group and greatly decreased in tumors with *NDFIP1* overexpression (Figs. 5A, 5B, S14A, and S14B), which is unrelated to the *TAZ* mRNA level (Fig. 5C and 5D). And cellular TAZ was negatively correlated with NDFIP1 in tumor tissues (Fig. 5E).

Moreover, the TAZ in equal number of exosomes from serum of *NDFIP1* knockout group mice was lower than that from control mice serum (Fig. 5F). In contrast, higher levels of TAZ were detected in exosomes from serum of *NDFIP1* overexpression group mice compared with the control group (Fig. 5G). In addition, exosomal TAZ was positively correlated with NDFIP1 in tumor tissues (Fig. 5H), and the TAZ in tumor cells had a negative correlation with the exosomal TAZ (Fig. 5I), indicating that NDFIP1 acted as the molecular switch of intra- and extra-cellular TAZ.

### Negative correlation between TAZ and NDFIP1 expression in NSCLC patient samples, and exosomal TAZ level is lower in the serum from NSCLC patients

**Figure 6.**
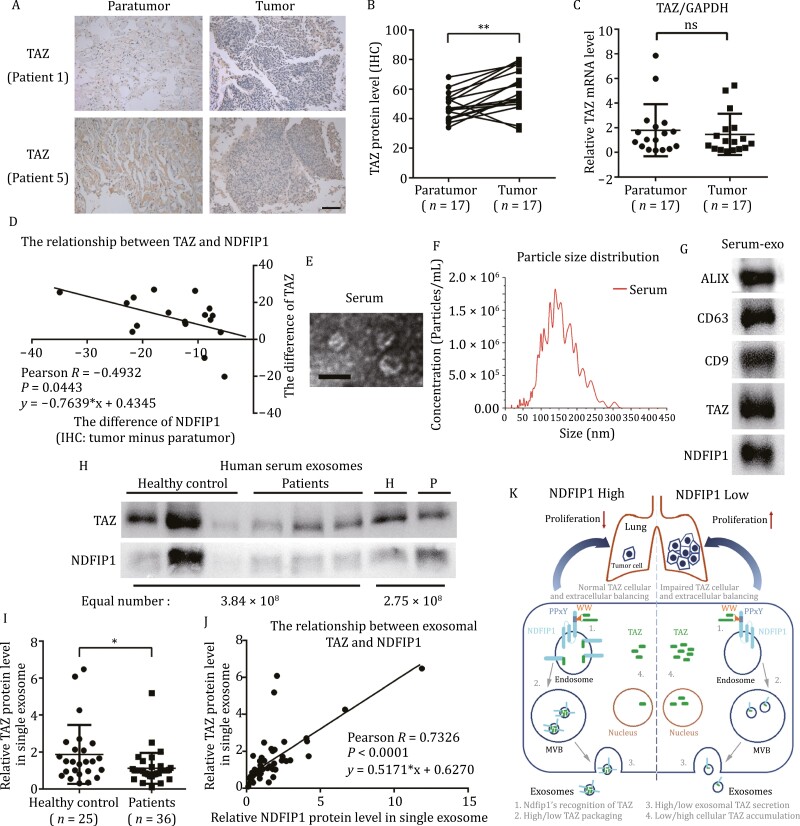
**Negative correlation between TAZ and NDFIP1 expression in NSCLC patient samples, and the level of TAZ in circulating exosomes.** (A) IHC of TAZ in tumor and paratumor tissues from patients 1 and 5. Scale bars: 200 μm. (B) Quantification of IHC staining intensity for TAZ in 17 paired NSCLC tissues. **P < 0.01. (C) TAZ mRNA levels in 17 NSCLC tissues and matched non-tumor tissues (*n* = 17). Bars represent the mean ± SD. ns, *P* = 0.5850. (D) Linear correlation analysis of relative TAZ level with relative NDFIP1 level in NSCLC tumor tissues compared with paratumor tissues (*n* = 17). (E) TEM image of exosomes from human serum. Scale bars: 100 nm. (F) The particle size distribution of exosomes from human serum. (G) The protein levels of YAP, TAZ, cTAZ, NDFIP1 and exosome-specific proteins, ALIX, CD63, CD9 in exosomes from human serum. (H) The TAZ and NDFIP1 protein levels in equal number of serum exosomes from the healthy control (H) and NSCLC patients (P). H = healthy control, P = patients. (I) Relative quantification of TAZ protein level in single exosome of healthy control (*n* = 25) and NSCLC patients (*n* = 36). Bars represent the mean ± SD. *P < 0.05. (J) Linear correlation analysis of TAZ with NDFIP1 in single exosome from human serum (*n* = 61). (K) Proposed model for the mechanism of lung cancer cell proliferation involving the export of TAZ via exosomes, in which the sorting of TAZ is controlled by NDFIP1. After recognition by NDFIP1, TAZ is packaged into exosomes; the increase of exosomal TAZ leads to the decrease of TAZ in cell cytoplasm and nucleus and tumor cell proliferation suppression.

To determine the clinical relevance of NDFIP1 and TAZ, we further analyzed the TAZ level in the same 17 pairs of samples from NSCLC patients. Compared with matched paratumor tissues, the TAZ protein levels were mostly upregulated in tumor tissues (Figs. 6A, 6B and S15). However, tumor samples did not exhibit a higher mRNA level of TAZ (Fig. 6C). Thus, the higher TAZ protein level in tumor tissues was also not caused by the increased transcriptional level of TAZ. Moreover, the protein level of TAZ and NDFIP1 exhibited a negative relationship (Fig. 6D).

Furthermore, we investigated the exosomal TAZ level in clinical samples. First, we isolated exosomes from human sera by ultracentrifugation method and characterized them using TEM, NTA, and Western blot (Fig. 6E–G). Then we examined the TAZ level in equal number of exosomes from 25 healthy subjects and 36 NSCLC patients (Figs. 6H and S16). Compared with the healthy subjects, lower TAZ levels were detected in exosomes from NSCLC patients (Fig. 6I). Expectedly, the single exosomal TAZ level had a positive correlation with NDFIP1 (Fig. 6J).

Taken together, the negative correlation between the expression of NDFIP1 and TAZ was verified in NSCLC clinical samples, and lower serum exosomal TAZ could be used for NSCLC diagnosis.

## Discussion

This study has identified a new tumor suppressor, NDFIP1 in NSCLC, and its novel function in controlling the quantity of cellular and exosomal TAZ. In the cells with higher NDFIP1 levels, more NDFIP1 effectively binds with more TAZ, and thus recruits more TAZ into exosomes, leading to lower levels of TAZ in both the cytoplasm and the nucleus, and ultimately less cell proliferation; on the contrary, in the tumor cells with lower NDFIP1 levels, the balance of cellular and extracellular TAZ is switched otherwise, that is, less TAZ is recognized by NDFIP1 and packaged into exosomes, resulting in more TAZ remaining in both the cytoplasm and the nucleus, and more cell proliferation (summarized in the schematic drawing, Fig. 6K). In the light of the findings in this study, low NDFIP1 level in tumor tissues and low TAZ level in single serum exosome may serve as diagnostic indexes for NSCLC, although a larger sample size is needed in the future study.

TAZ, also known as WWTR1, has been identified to drive tumor formation, survival, stemness, progression, metastasis, and resistance to therapy; and TAZ overexpression has been associated with development, progression, and poor prognosis in NSCLC. However, increased expression of TAZ is hardly dependent of genomic amplification of *TAZ* loci, but instead depends on the cell-autonomous genetic/epigenetic alterations of TAZ upstream regulators in tumor cells or on the non-cell-autonomous mechanical/biochemical changes occurring in the tumor microenvironment (Lo Sardo et al., 2018). In this study, we first revealed that NDFIP1 negatively regulates TAZ by sorting TAZ into exosomes, expanding the understanding of the regulatory mechanisms on TAZ.

Furthermore, we have shown for the first time that NDFIP1 inhibits tumor cell proliferation in NSCLC through the regulation of TAZ. Previously, NDFIP1 has been reported as a tumor suppressor in other tumor types and can be downregulated by upstream miRNAs or external stimuli such as hypoxia and nicotine (Peng et al., 2017; Zhang et al., 2019; Ben et al., 2020). However, the researches on the underlying mechanism of NDFIP1 repressing cell proliferation were limited. In one study, miR-873 activated the key glycolytic proteins AKT/mTOR via targeting NDFIP1 to promote hepatocellular carcinoma growth and metastasis (Zhang et al., 2019). In the other study, genetic deletion of *NDFIP1* resulted in a loss of PTEN nuclear compartmentalization and increased cell proliferation in a human neuroblastoma cell line SH-SY5Y (Howitt et al., 2015). So, our data not only extend the tumor-suppressing function of NDFIP1 to NSCLC, but also provide a new downstream mechanism related to cellular and exosomal TAZ.

Exosomes are known as the mediators of intercellular communication. Especially in cancers, exosomes are commonly called “Oncosomes” because the secreted exosomes that contain oncogenic cargos could target other cells in the primary tumor microenvironment and the distant premetastatic niche to promote tumorigenesis and development (Rak and Guha, 2012; Costa-Silva et al., 2015; Zhang et al., 2015; Becker et al., 2016). Nevertheless, what happens in the donor cells after exosomes secretion has attracted little attention until recently. Indeed, exosomes were discovered as a way to expel waste in 1980s (Harding et al., 1983), and this originally identified function of exosomes should not be ignored (Chairoungdua et al., 2010; Mc Namee and O’Driscoll, 2018; Han et al., 2019; Majer et al., 2019; Miao *et al.*, 2015; Strzyz, 2020). For instance, colon tumor cells are inclined to selectively sort tumor suppressor miRNAs into exosomes and retain more oncogenic miRNAs to promote tumor progression (Teng et al., 2017). Moreover, *ALIX* depletion in breast cancer cells resulted in decreased exosomal PD-L1, followed by higher PD-L1 surface presentation and increased immunosuppression (Monypenny et al., 2018). Here, we found NSCLC cells downregulate NDFIP1 to keep more intracellular TAZ from being secreted via exosomes, providing new evidences of how exosomes affect their donor cells.

Exosomes originate from inner budding of late endosomes, followed by the membrane fusion of multivesicular bodies (MVBs) and the plasma membrane (Shao et al., 2018). The formation of MVBs involves late-domain (L-domain) proteins, ubiquitin, and the endosomal sorting complex required for transport (ESCRT) machinery (Sterzenbach et al., 2017). Containing two PPxY motifs, NDFIP1 is an adaptor of Nedd4 family for ubiquitination; so, it is not surprising that NDFIP1 has been identified to interact with target proteins, leading to their ubiquitination and exosomal packaging (Harvey et al., 2002; Putz et al., 2008, 2012; Sterzenbach et al., 2017). In this study, we first reported the interaction of TAZ with NDFIP1 and subsequent exosomal loading. Given that YAP and TAZ possess similar structural features including WW domain(s), it is interesting that although both YAP and TAZ are detectable in cell lysates, a polyclonal antibody to NDFIP1 pulled down TAZ, but not YAP (Fig. 2A). In fact, despite the consistency of structure and function, YAP and TAZ have distinct functions mediated by different protein–protein interactions (Cui et al., 2003; Hong et al., 2005; Callus et al., 2019). In humans, YAP isoforms contain either one (YAP1) or two (YAP2) WW domains, whereas TAZ isoforms contain only a single WW domain. Several studies consider that with two WW domains, YAP/TAZ isoforms possess higher affinity for multi-PPxY partner proteins (Callus et al., 2019). In contrast, another study showed the negative cooperation between the tandem WW domains of YAP2 when binding to their cognate ligands (Schuchardt et al., 2014). Hence, WW domain–PPxY interactions may be much more complex, and therefore current knowledge is inadequate to explain the different NDFIP1 interaction patterns between YAP and TAZ.

In conclusion, NDFIP1 plays an essential role in the recruitment of TAZ into exosomes, therefore balancing the levels of intracellular and extracellular TAZ; NDFIP1, as a tumor suppressor, is downregulated in NSCLC, therefore releasing the brake of cell proliferation inhibition. Thus, the new function of NDFIP1 and the new regulatory mechanism of TAZ, uncovered in this study, have implications for the development of biomarkers and treatment strategies of NSCLC.

## Materials and methods

### Human tissue and blood samples collection

Seventeen pairs of NSCLC tumor and paratumor tissue samples as well as 61 blood samples (25 for normal donors and 36 for NSCLC patients) were obtained from Shanghai Jiao Tong University-affiliated Shanghai Chest Hospital after surgical resection. The tissue samples were next subjected to IHC and PCR experiments. And blood samples were centrifuged at 3,000 rpm at 4°C for 10 min for serum collection, followed by exosomes isolation.

This study was approved by the Ethical Committee of the School of Biomedical Engineering, Shanghai Jiao Tong University and carried out in accordance with the Declaration of Helsinki. All participants were provided written informed consents.

### Cell culture

HEK-293T, Beas-2B, A549, SPC-A1, SPC-A1-BM cells were cultured in Dulbecco’s Modified Eagle’s Medium (DMEM; HyClone, SH30243.01) while H520, H1581, HCC95, and H2170 cells were cultured in Roswell Park Memorial Institute (RPMI; HyClone, SH30809.01) containing 10% fetal bovine serum (FBS; LONSA, S711-001S) and 1% penicillin-streptomycin (PS; HyClone, SV30010). Cells were incubated at 37°C in a 5% CO_2_ incubator (Thermo, Forma Series II) for follow-up experiments. SPC-A1-BM is a highly bone metastatic cell line established from SPC-A1 by *in vivo* selection in BALB/c mouse models (Yang et al., 2009; Yu et al., 2014; He et al., 2019).

To deplete bovine exosomes from FBS, FBS was diluted to 20% by conditioned medium and then centrifuged at 120,000 ×*g* for 16 h at 4°C. For exosomes isolation, cultures were incubated with exosome-free FBS for 24 h prior to collecting the cell culture medium.

### Exosomes isolation

Exosomes from serum and cell conditioned media were both isolated by ultracentrifugation method. Briefly, the serum was first centrifuged 15 min at 3,000 ×*g*, 4°C, followed by 30 min, 10,000 ×*g*. Then, the supernatant was transferred into a 6-mL ultracentrifuge tube (Beckman, 344619). The tube was then filled with PBS (HyClone, SH30256.01) and ultracentrifugation was done twice for 70 min at 100,000 ×*g*, 4°C in a Type 100 Ti swinging-bucket rotor (Beckman). As for conditioned media, it was first centrifuged for 5 min at 500 ×*g*, 4 ×*g*, followed by for 30 min at 2,000 ×*g*, and for 35 min at 10,000 ×*g*. Then, the supernatant was transferred into 38.5 mL ultracentrifuge tubes (Beckman, 326823) and ultracentrifuged twice for 70 min at 100,000 ×*g*, 4°C, in an SW 32 Ti swinging-bucket rotor (Beckman). The deposits in each step are, respectively, referred to as intact cells in suspension during cell culture, cell debris, extracellular vesicles (EVs), and exosomes as shown in Fig. S5A. For exosomal TAZ level comparison, a filtration process by a 0.22 μm filter was performed to exclude particles >200 nm before ultracentrifugation (Lobb et al., 2015; Jeppesen et al., 2019).

### Transmission electron microscopy

The exosome pellet was resuspended in PBS for transmission electron microscopy (TEM; FEI, Tecnai G2 spirit Biotwin). Briefly, 10 μL of exosomes was dripped onto a copper grid (Zhongjingkeyi, CHN, BZ110223b). After 1 min of sedimentation, the droplet was sucked out using the air-laid paper. And then, 10 μL of 2% uranyl acetate (Merck, 1005) solution was dripped onto the same copper grid for negative staining and sucked out again 1 min later.

### Nanoparticle tracking analysis (NTA)

The size distribution of exosomes was measured by ZetaView (Particle Metrix). In brief, the exosome pellet was resuspended in a proper volume of PBS to achieve the optimal detectable concentration (about 10^7^ particles/mL). For each measurement, 3–5 mL of the diluted sample was injected into the instrument, and the concentration of this sample as well as the size distribution were measured by the machine software (ZetaView 8.03.04.01).

### Western blot

Western blotting was performed to detect the protein level of different samples. Generally, total protein was extracted by lysis buffer (RIPA, Millipore, 20-188) containing protease inhibitor cocktail (CWBIO, 2200S), phosphatase inhibitor (CWBIO, 2383S), and phenylmethyl sulfonyl fluoride (PMSF, Beyotime, ST506-2). And then the protein concentration was measured using a BCA assay kit (Thermo, 23227). As for exosomal TAZ level comparison, equal number of exosomes measured by NTA was used for Western blotting. Each sample with same amount of total proteins or exosomes was mixed with a loading buffer (5×) and heated at 95°C for 5 min. The denatured proteins were loaded on 10% sodium dodecyl sulfate-polyacrylamide gel (SDS-PAGE; EpiZyme, PG212) and separated at constant 120 V for 80 min. Then, proteins were transferred to a nitrocellulose membrane (GE Healthcare, 10600002) at constant 300 mA for 1.5 h. The membrane was blocked with 5% nonfat milk powder suspended in Tris-buffered saline and Tween 20 (TBST) for 1 h at room temperature. The blots were probed with primary antibodies: TSG101 (Abcam, 133586), HSP70 (CST, 4872S), ALIX (CST, 2171S), CD63 (Santa, 5275), CD9 (Abcam, 92726), CD81 (Santa, 166029), GM130 (R&D, 81991), RAB27B (Abcam, 103418), YAP/TAZ (CST, E9M8G), TAZ (CST, E8E9G), NDFIP1 (Santa, 398469), β-Tubulin (Abcam, 6046), GAPDH (Proteintech, 10494), Lamin A/C (Abcam, 108595), PCNA (Proteintech, 10205), and these primary antibodies were subsequently probed with appropriate horseradish peroxidase conjugated secondary antimouse or rabbit antibodies (Jackson, 115-035-003 or 111-035-003). Finally, the blots were visualized using the enhanced chemiluminescence (ECL; Thermo, 1856136) and chemiluminescence imaging system (Tanon, 5200). The intensity of each band was analyzed by ImageJ software.

### Immunofluorescence

Immunofluorescence (IF) was performed for co-localization. Generally, cells were seeded on coverslips in a 24-well plate and cultured, then fixed with 4% paraformaldehyde for 10 min at room temperature. After fixation, cells were rinsed three times with PBS followed by permeabilizing in 0.5% Triton X-100 for 10 min and blocking in 1% bovine serum albumin (BSA) in PBS for 1 h. And then cells were incubated with primary antibody (CD63, Santa, 5275; TAZ, CST, E8E9G; NDFIP1, Santa, 398469) at 1:200 dilutions at 4°C overnight. Alternatively, cells were incubated with rabbit IgG conjugated with Alexa Fluor 488 (Invitrogen, A21206) and mouse IgG conjugated with Alexa Fluor 594 (Invitrogen, A21203) or mouse IgG conjugated with Alexa Fluor 488 (Invitrogen, A21202) and rabbit IgG conjugated with Alexa Fluor 594 (Invitrogen, A21207). Cell nuclei were stained with DAPI (Beyotime, C1002) for 5 min at room temperature. Stained cells were photographed under an immunofluorescence microscope (Leica, DFC420C) or a confocal microscope (Leica, TCS SP5 II) and qualified with ImageJ software.

### Co-immunoprecipitation

Co-immunoprecipitation (Co-IP) was performed according to the manufacturer’s instruction (Capturem IP & Co-IP Kit, Takara, 635721). Briefly, cells grown in 10-cm dishes were washed once with PBS and lysed on ice for 15 min with 1-mL lysis buffer. Following 17,000 ×*g* centrifugation at 4°C for 10 min, the supernatant was divided into 200, 400, and 400 μL (Fig. S2A), and the two portions of 400 μL were incubated with 8 μg antibody (Normal Rabbit IgG, CST, 2729S; TAZ, CST, E8E9G; Normal Mouse IgG, Santa, 2025; NDFIP1, Santa, 398469) for 1 h at 4°C. After incubation, 400 μL sample was added onto the spin column and centrifuged at 1000 ×*g* for 1 min at room temperature. Then, 100 μL wash buffer was added to the spin column and centrifuged at 1000 ×*g* for 1 min again. Finally, 30 μL elution buffer was added to the column and centrifuged at 1000 ×*g* for 1 min. The eluted sample is now ready for Western blot analysis.

### Public dataset analysis from Oncomine and Kaplan-Meier Plotter

Relative copy number and mRNA levels of NDFIP1 in different lung cancer datasets were downloaded from Oncomine database and analyzed using the Graphpad software. Kaplan-Meier overall survival (OS) and progression-free survival (PFS) curve of NSCLC patients with low or high expression of NDFIP1 was generated using Kaplan–Meier Plotter (Gyorffy et al., 2014).

### Immunohistochemistry

Paraformaldehyde (4%)-fixed tissues were embedded in paraffin followed by sectioning (5 μm in thickness) with a microtome (Leica, Solms, RM2245). After xylene dewaxing, PFA fixation, 0.3% Triton X-100 permeabilization, tissues were blocked with 10% goat serum for 1 h at room temperature and incubated with primary antibodies (TAZ, CST, E9J5A; NDFIP1, Santa, 398469; Ki-67, Proteintech, 27309) at 4°C overnight. After three times washing, tissues were incubated with secondary antibodies (Jackson, 115-035-003; Jackson, 111-035-003; Invitrogen, A21207) for 1 h at room temperature. Diaminobenzidine hydrogen peroxide (Sigma) was the chromogen, and the counterstaining was carried out with 0.5% hematoxylin. The intensity of immunohistochemistry (IHC) staining was qualified by an IHC Profiler in ImageJ software.

### Quantitative real-time PCR (qRT-PCR)

Total RNA was first extracted from cells with RNAiso Plus reagent (Takara, 9109). And then PrimerScript reverse transcriptase (RT) reagent kit with gDNA Eraser (Takara, RR047A) was used to synthesize cDNA from total RNA. Briefly, total RNA diluted by RNase-free ddH_2_O was mixed with 5× gDNA eraser buffer and gDNA eraser at 42°C for 2 min to remove gDNA. Secondly, the sample was mixed with 5× PrimeScript Buffer 2, PrimeScript RT Enzyme Mix 1, and RT Primer Mix at 37°C for 15 min followed by 85°C for 5 s and 4°C forever. Finally, quantitative real-time PCR was performed on a Fast Real-Time PCR System (ABI, 7900HT) by using TB Green Premix Ex TaqTM II (Takara, RR820A). Briefly, the synthesized cDNA was mixed with ROX Reference Dye II, TB green Premix Ex Taq II, and forward and reverse primer at 95°C for 30 s, followed by 40 cycles of 95°C for 5 s, 60°C for 34 s, and ending at 95°C for 15 s, 60°C for 1 min, and 95°C for 15 s. The sequence of each primer used was summarized as follows: GAPDH-F is 5ʹ-GGAGCGAGATCCCTCCAAAAT-3ʹ; GAPDH-R is 5ʹ-GGCTGTTGTCATACTTCTCATGG-3ʹ; NDFIP1-F is 5ʹ-CCAGCTGAGGATAGGAAACG-3ʹ; NDFIP1-R is 5ʹ-GGCATCTTCCGAACTTTTGC-3ʹ; TAZ-F is 5ʹ-CACCCGGCCTTGATGTTTAT-3ʹ; TAZ-R is 5ʹ-TGTGTGGTGTGCTATCAGGT-3ʹ.

### Lentivirus packaging and stable cell lines generation

Human *NDFIP1* knockdown plasmid (shNDFIP1) and control plasmid (shNC), *NDFIP1* overexpression plasmid (NDFIP1-OE) and negative control plasmid (PCDH) were purchased from OBIO (Shanghai, China). Human *NDFIP1* knockout Crispr-cas9 plasmid (sgNDFIP1) and negative control plasmid (sgNC) were purchased from ZORINBIO (Shanghai, China). To generate the lentivirus containing plasmid, HEK 293T cells were co-transfected with VSVG, pDelta, and the indicated plasmid. And the concentrated lentivirus was obtained from conditioned medium after ultracentrifugation. For infection, the viral stock was added into SPC-A1 cells with 7 μg/mL polybrene. NDFIP1-OE and PCDH GFP^+^ cells can be subsequently selected by fluorescence-activated cell sorting (FACS; BD, FACS Aria II) while sgNDFIP1 and sgNC cells need to be selected by 1 μg/mL of puromycin with a monoclonal cultivation by inoculating into a 96-well plate.

Lentiviruses expressing shRNA were purchased from OBIO (Shanghai, China). The sequence of the shRAB27B was 5ʹ-CCCAAATTCATCACTACAGTA-3ʹ.

### Nuclear and cytoplasmic protein extraction

Nuclear and cytoplasmic protein extraction were performed according to the manufacturer’s instruction (Nuclear and Cytoplasmic Protein Extraction Kit, Beyotime, P0028). Briefly, cells grown in a 24-well plate were washed once with PBS and scraped for centrifugation to obtain cell pellet. Then 200 μL cytoplasmic protein extraction buffer A was added into cell pellet and vortexed for several seconds. After 15 min, 10-μL cytoplasmic protein extraction buffer A was added and vortexed for several seconds followed by centrifugation at 12,000 ×*g* for 5 min. At this point, the supernatant is the cytoplasmic fraction. Then 50-μL nuclear protein extraction buffer was added into the pellet followed by an intermittent vortex for 30 min and centrifugation at 12,000 ×*g* for 10 min. The resultant supernatant is the nuclear fraction.

### Drug treatment

Cells were seeded in a 6-well plate in the absence or presence of drugs. For degradation rate assessment, 12.5 μg/mL of cycloheximide (CHX, MCE, 12320) was added for 1–6 h. For the inhibition of lysosome or proteasome-mediated degradation pathway, 5 μmol/L MG132 (MCE, 13259) or 50 μmol/L chloroquine (CQ, MCE, 17589A) was added for 24 h. To inhibit exosome secretion, 5 μmol/L GW4869 (MCE, HY-19363) was added for 24 h.

### siRNA and plasmid transfection

Cells were transfected with siRNAs using RNAFit Reagent (HanBio, HB-RF-1000) according to the manufacturer’s protocol. Briefly, the cells were incubated with the complex of siRNA and RNAFit reagent, and the gene knockdown efficiency was assessed after 48 h. siRNAs was synthesized by RiboBio. The siTAZ sequence was 5ʹ-GGTACTTCCTCAATCACAT-3ʹ.

Cells were transfected with plasmids using Lipofectamine 3000 (Thermo, L3000015) according to the manufacturer’s protocol. Briefly, the cells were incubated with the complex of plasmids, P3000 and Lipofectamine 3000 reagent, and the plasmid transfection efficiency was assessed after 48 h. The TAZ WT (3XFlag pCMV5-TOPO TAZ WT, 24809) and TAZ ΔWW (3XFlag pCMV5-TOPO TAZ (∂WW), 24811) plasmids were purchased from Addgene.

### Cell counting kit-8 (CCK-8)

For the proliferation assay, the cell viability at different times (every 24 h) was examined by CCK-8 assay kit (Target Mol, C0005). Briefly, different stable expression cells were seeded in five 96-well plates at a density of 2,000 cells per well (*n* = 6). As for siTAZ interference, different stable expression cells were seeded in three 96-well plates at a density of 5,000 cells per well (*n* = 6) at 2 days after transfection. Every 24 h, 10 μL of CCK8 solution was added to each well in one plate and incubated for 90 min at 37°C. The OD value at 450 nm was measured by the microplate reader (BioTek, Synergy2).

### Tumor xenograft model

BALB/c nude mice (*n* = 6) were subcutaneously injected into the right flanks with a total of 5 × 10^6^ stably transfected SPC-A1 cells in a volume of 50 µL. Tumor sizes and body weights were monitored every 3 days. All the mice were housed in the specific pathogen-free animal room of Shanghai Jiao Tong University. At day 21, mice were sacrificed and the blood samples were isolated by eyeball extirpating. Meanwhile, tumors were harvested and cut into three pieces for protein extraction, RNA extraction, and paraffin embedding.

This study was approved by the Ethical Committee of the School of Biomedical Engineering, Shanghai Jiao Tong University, and all procedures were performed following the regulations and internal biosafety and bioethics guidelines of Med-X Research Institute, Shanghai Jiao Tong University.

### Statistical analysis

All statistical analyses were performed using the GraphPad Prism 6 software. Data are presented as mean ± SD, and the paired or unpaired *t*-test or ANOVA were chosen to analyze the statistical significance. The probability value below 0.05 was considered significant.

## Supplementary Material

pwac017_suppl_Supplementary_Material

## Abbreviations

ALIX: apoptosis-linked gene 2 (ALG-2) interacting protein X
ANOVA: analysis of variance
BCA: bicinchoninic acid
BSA: bovine serum albumin
CCK: cell counting kit
CD9: cluster of differentiation 9
CD63: cluster of differentiation 63
CD81: cluster of differentiation 81
CHX: cycloheximide
co-IP: co-immunoprecipitation
CQ: chloroquine
DAPI: fluorescence-activated cell sorting
DMEM: Dulbecco’s modified eagle’s medium
ECL: enhanced chemiluminescence
EMT: epithelial mesenchymal transition
ESCRT: endosomal sorting complex required for transport
EV: extracellular vesicles
FACS: fluorescence-activated cell sorting
FBS: fetal bovine serum
GAPDH: Glyceraldehyde-3-phosphate dehydrogenase
GFP: green fluorescent protein
GM130: Golgi matrix protein 130
HSP70: heat shock protein 70
IF: immunofluorescence
IHC: immunohistochemistry
IgG: immunoglobulin G
LADC: lung adenocarcinoma
LATS1/2: large tumor suppressor gene 1/2
L-domain: late-domain
LLC: lung large cell carcinoma
LSQ: lung squamous cell carcinoma
miRNA: microRNA
MST1/2: mammalian sterile 20-like kinase 1/2
MVB: multivesicular bodies
NDFIP1: Nedd4 family-interacting protein 1
NSCLC: non-small cell lung cancer
NTA: nanoparticle tracking analysis
OD: optical density
OS: overall survival
PCNA: proliferating cell nuclear antigen
PCR: polymerase chain reaction
PFS: progression-free survival
PMSF: phenylmethyl sulfonyl fluoride
PS: penicillin-streptomycin
PTEN: phosphatase and tensin homolog deleted on chromosome ten
RPMI: Roswell Park Memorial Institute
SD: standard deviation
SDS-PAGE: sodium dodecyl sulfate polyacrylamide gel
SLC: small cell Lung cancer
TAZ: Transcriptional coactivator with PDZ-binding motif
TBST: Tris-buffered saline and Tween 20
TEM: transmission electron microscopy
TSG101: tumor susceptibility gene 101
WWTR1: WW domain-containing transcription regulator 1
YAP: yes associated protein

## Declarations

This work was supported by grants from Science and Technology Commission of Shanghai Municipality (No. 21ZR1433100), State Key Laboratory of Oncogenes and Related Genes (No. KF2122), National Natural Science Foundation of China (No. 81773115), and Shanghai Jiao Tong University Interdisciplinary Research Funding (Nos. YG2022ZD016, YG2017MS52).

This study was approved by the Ethical Committee of the School of Biomedical Engineering, Shanghai Jiao Tong University (Nos. 2019045 and 2020020).

Y.C. conceived and designed the experiments, performed the experiments, analyzed the data, prepared figures, and wrote drafts of the paper. X.L., Z. C., Y.Z., and T.W. performed the experiments and analyzed the data. F.L., Q.H., and Q.Z. conceived and designed the experiments, and analyzed the data. Z.L., S.L. provided the clinical-related materials and supported related analysis. C.W. provided guidance in experiment design and was involved in writing of the draft. W.X. conceived and designed the experiments, reviewed drafts of the paper, and approved the final draft.

## References

[CIT0001] Banik SM , Pedram K, Wisnovsky S et al. Lysosome-targeting chimaeras for degradation of extracellular proteins. Nature 2020;584:291–297. 32728216 10.1038/s41586-020-2545-9 PMC7727926

[CIT0002] Becker A , Thakur BK, Weiss JM et al. Extracellular vesicles in cancer: cell-to-cell mediators of metastasis. Cancer Cell 2016;30:836–848. 27960084 10.1016/j.ccell.2016.10.009 PMC5157696

[CIT0003] Ben QW , Sun YW, Liu J et al. Nicotine promotes tumor progression and epithelial-mesenchymal transition by regulating the miR-155-5p/NDFIP1 axis in pancreatic ductal adenocarcinoma. Pancreatology 2020;20:698–708. 32354626 10.1016/j.pan.2020.04.004

[CIT0004] Callus BA , Finch-Edmondson ML, Fletcher S et al. YAPping about and not forgetting TAZ. FEBS Lett 2019;593:253–276. 30570758 10.1002/1873-3468.13318

[CIT0005] Chairoungdua A , Smith DL, Pochard P et al. Exosome release of beta-catenin: a novel mechanism that antagonizes Wnt signaling. J Cell Biol 2010;190:1079–1091. 20837771 10.1083/jcb.201002049 PMC3101591

[CIT0006] Chen YA , Lu CY, Cheng TY et al. WW domain-containing proteins YAP and TAZ in the Hippo pathway as key regulators in stemness maintenance, tissue homeostasis, and tumorigenesis. Front Oncol 2019;9:60. 30805310 10.3389/fonc.2019.00060 PMC6378284

[CIT0007] Ciechanover A. Proteolysis: from the lysosome to ubiquitin and the proteasome. Nat Rev Mol Cell Biol 2005;6:79–86. 15688069 10.1038/nrm1552

[CIT0008] Costa-Silva B , Aiello NM, Ocean AJ et al. Pancreatic cancer exosomes initiate pre-metastatic niche formation in the liver. Nat Cell Biol 2015;17:816–826. 25985394 10.1038/ncb3169 PMC5769922

[CIT0009] Cui CB , Cooper LF, Yang X et al. Transcriptional coactivation of bone-specific transcription factor Cbfa1 by TAZ. Mol Cell Biol 2003;23:1004–1013. 12529404 10.1128/MCB.23.3.1004-1013.2003 PMC140696

[CIT0010] Fang C , Li J, Qi S et al. An alternatively transcribed TAZ variant negatively regulates JAK-STAT signaling. EMBO Rep 2019;20(6):e47227. 30979708 10.15252/embr.201847227 PMC6549033

[CIT0011] Gorla M , Santiago C, Chaudhari K et al. Ndfip proteins target Robo receptors for degradation and allow commissural axons to cross the midline in the developing spinal cord. Cell Rep 2019;26:3298–3312.e4. 30893602 10.1016/j.celrep.2019.02.080 PMC6913780

[CIT0012] Gyorffy B , Surowiak P, Budczies J et al. Online survival analysis software to assess the prognostic value of biomarkers using transcriptomic data in non-small-cell lung cancer (vol 8, e82241, 2013). PLoS One 2014;9:ARTN e111842. 10.1371/journal.pone.0082241 PMC3867325 24367507

[CIT0013] Han Q , Lv L, Wei J et al. Vps4A mediates the localization and exosome release of beta-catenin to inhibit epithelial-mesenchymal transition in hepatocellular carcinoma. Cancer Lett 2019;457:47–59. 31059752 10.1016/j.canlet.2019.04.035

[CIT0014] Harding C , Heuser J, Stahl P. Receptor-mediated endocytosis of transferrin and recycling of the transferrin receptor in rat reticulocytes. J Cell Biol 1983;97:329–339. 6309857 10.1083/jcb.97.2.329 PMC2112509

[CIT0015] Harvey KF , Shearwin-Whyatt LM, Fotia A et al. N4WBP5, a potential target for ubiquitination by the Nedd4 family of proteins, is a novel golgi-associated protein. J Biol Chem 2002;277:9307–9317. 11748237 10.1074/jbc.M110443200

[CIT0016] He S , Li Z, Yu Y et al. Exosomal miR-499a-5p promotes cell proliferation, migration and EMT via mTOR signaling pathway in lung adenocarcinoma. Exp Cell Res 2019;379:203–213. 30978341 10.1016/j.yexcr.2019.03.035

[CIT0017] Herbst RS , Morgensztern D, Boshoff C. The biology and management of non-small cell lung cancer. Nature 2018;553:446–454. 29364287 10.1038/nature25183

[CIT0018] Hong JH , Hwang ES, McManus MT et al. TAZ, a transcriptional modulator of mesenchymal stem cell differentiation. Science 2005;309:1074–1078. 16099986 10.1126/science.1110955

[CIT0019] Howitt J , Low LH, Putz U et al. Ndfip1 represses cell proliferation by controlling Pten localization and signaling specificity. J Mol Cell Biol 2015;7:119–131. 25801959 10.1093/jmcb/mjv020

[CIT0020] Howitt J , Putz U, Lackovic J et al. Divalent metal transporter 1 (DMT1) regulation by Ndfip1 prevents metal toxicity in human neurons. Proc Natl Acad Sci USA 2009;106:15489–15494. 19706893 10.1073/pnas.0904880106 PMC2741278

[CIT0021] Jeppesen DK , Fenix AM, Franklin JL et al. Reassessment of exosome composition. Cell 2019;177:428–445.e18. 30951670 10.1016/j.cell.2019.02.029 PMC6664447

[CIT0022] Lee DH , Goldberg AL. Proteasome inhibitors: valuable new tools for cell biologists. Trends Cell Biol 1998;8:397–403. 9789328 10.1016/s0962-8924(98)01346-4

[CIT0023] Lewis DR , Check DP, Caporaso NE et al. US lung cancer trends by histologic type. Cancer 2014;120:2883–2892. 25113306 10.1002/cncr.28749 PMC4187244

[CIT0024] Liu Y , Zhang X, Lin JF et al. CCT3 acts upstream of YAP and TFCP2 as a potential target and tumour biomarker in liver cancer. Cell Death Dis 2019;10:ARTN 644. 10.1038/s41419-019-1894-5 PMC6733791 31501420

[CIT0025] Lo Sardo F , Strano S, Blandino G. YAP and TAZ in lung cancer: oncogenic role and clinical targeting. Cancers (Basel) 2018;10(5):137. 29734788 10.3390/cancers10050137 PMC5977110

[CIT0026] Lobb RJ , Becker M, Wen SW et al. Optimized exosome isolation protocol for cell culture supernatant and human plasma. J Extracell Vesicles 2015;4:ARTN 27031. 10.3402/jev.v4.27031 PMC4507751 26194179

[CIT0027] Lu T , Li Z, Yang Y et al. The Hippo/YAP1 pathway interacts with FGFR1 signaling to maintain stemness in lung cancer. Cancer Lett 2018;423:36–46. 29452146 10.1016/j.canlet.2018.02.015

[CIT0028] Ma F , Ding MG, Lei YY et al. SKIL facilitates tumorigenesis and immune escape of NSCLC via upregulating TAZ/autophagy axis. Cell Death Dis 2020;11:ARTN 1028. 10.1038/s41419-020-03200-7 PMC7710697 33268765

[CIT0029] Majer O , Liu B, Kreuk LSM et al. UNC93B1 recruits syntenin-1 to dampen TLR7 signalling and prevent autoimmunity. Nature 2019;575:366–370. 31546246 10.1038/s41586-019-1612-6 PMC6856441

[CIT0030] Mc Namee N , O’Driscoll L. Extracellular vesicles and anti-cancer drug resistance. Bba-Rev Cancer 2018;1870:123–136. 10.1016/j.bbcan.2018.07.003 30003999

[CIT0031] Menck K , Sonmezer C, Worst TS et al. Neutral sphingomyelinases control extracellular vesicles budding from the plasma membrane. J Extracell Vesicles 2017;6:1378056. 29184623 10.1080/20013078.2017.1378056 PMC5699186

[CIT0032] Miao YX , Li GJ, Zhang XL et al. A TRP channel senses lysosome neutralization by pathogens to trigger their expulsion. Cell 2015;161:1306–1319. 26027738 10.1016/j.cell.2015.05.009 PMC4458218

[CIT0033] Monypenny J , Milewicz H, Flores-Borja F et al. ALIX regulates tumor-mediated immunosuppression by controlling EGFR activity and PD-L1 presentation. Cell Rep 2018;24:630–641. 30021161 10.1016/j.celrep.2018.06.066 PMC6077252

[CIT0034] O’Leary CE , Riling CR, Spruce LA et al. Ndfip-mediated degradation of Jak1 tunes cytokine signalling to limit expansion of CD4+ ­effector T cells. Nat Commun 2016;7:ARTN 11226. 10.1038/ncomms11226 PMC4837450 27088444

[CIT0035] Oliver PM , Cao X, Worthen GS et al. Ndfip1 protein promotes the function of itch ubiquitin ligase to prevent T cell activation and T helper 2 cell-mediated inflammation. Immunity 2006;25:929–940. 17137798 10.1016/j.immuni.2006.10.012 PMC2955961

[CIT0036] Ostrowski M , Carmo NB, Krumeich S et al. Rab27a and Rab27b control different steps of the exosome secretion pathway. Nat Cell Biol 2010;12:19–30; sup pp 11-13. 19966785 10.1038/ncb2000

[CIT0037] Peng J , Liu HL, Liu CH. MiR-155 promotes uveal melanoma cell proliferation and invasion by regulating NDFIP1 expression. Technol Cancer Res Treat 2017;16:1160–1167. 29333944 10.1177/1533034617737923 PMC5762084

[CIT0038] Pocaterra A , Romani P, Dupont S. YAP/TAZ functions and their regulation at a glance. J Cell Sci 2020;133:ARTN jcs230425. 10.1242/jcs.230425 31996398

[CIT0039] Putz U , Howitt J, Doan A et al. The tumor suppressor PTEN is exported in exosomes and has phosphatase activity in recipient cells. Sci Signaling 2012;5:ARTN ra70. 10.1126/scisignal.2003084 23012657

[CIT0040] Putz U , Howitt J, Lackovic J et al. Nedd4 family-interacting protein 1 (Ndfip1) is required for the exosomal secretion of Nedd4 family proteins. J Biol Chem 2008;283:32621–32627. 18819914 10.1074/jbc.M804120200

[CIT0041] Rak J , Guha A. Extracellular vesicles – vehicles that spread cancer genes. Bioessays 2012;34:489–497. 22442051 10.1002/bies.201100169

[CIT0042] Salah Z , Alian A, Aqeilan RI. WW domain-containing proteins: retrospectives and the future. Front Biosci-Landmrk 2012;17:331–348. 10.2741/3930 22201747

[CIT0043] Schuchardt BJ , Mikles DC, Hoang LM et al. Ligand binding to WW ­tandem domains of YAP2 transcriptional regulator is under negative cooperativity. FEBS J 2014;281:5532–5551. 25283809 10.1111/febs.13095 PMC4262544

[CIT0044] Shao H , Im H, Castro CM et al. New technologies for analysis of extracellular vesicles. Chem Rev 2018;118:1917–1950. 29384376 10.1021/acs.chemrev.7b00534 PMC6029891

[CIT0045] Sterzenbach U , Putz U, Low LH et al. Engineered exosomes as vehicles for biologically active proteins. Mol Ther 2017;25:1269–1278. 28412169 10.1016/j.ymthe.2017.03.030 PMC5474961

[CIT0046] Strzyz P. Iron expulsion by exosomes drives ferroptosis resistance. Nat Rev Mol Cell Biol 2020;21:4. 10.1038/s41580-019-0195-2 31748716

[CIT0047] Sung H , Ferlay J, Siegel RL et al. Global cancer statistics 2020: GLOBOCAN estimates of incidence and mortality worldwide for 36 cancers in 185 countries. CA Cancer J Clin 2021;71:209–249. 33538338 10.3322/caac.21660

[CIT0048] Teng Y , Ren Y, Hu X et al. MVP-mediated exosomal sorting of miR-193a promotes colon cancer progression. Nat Commun 2017;8:14448. 28211508 10.1038/ncomms14448 PMC5321731

[CIT0049] Tian Z , He W, Tang J et al. Identification of important modules and biomarkers in breast cancer based on WGCNA. Onco Targets Ther 2020;13:6805–6817. 32764968 10.2147/OTT.S258439 PMC7367932

[CIT0050] Trajkovic K , Hsu C, Chiantia S et al. Ceramide triggers budding of exosome vesicles into multivesicular Endosomes. Science 2008;319:1244–1247. 18309083 10.1126/science.1153124

[CIT0051] Varelas X , Sakuma R, Samavarchi-Tehrani P et al. TAZ controls Smad nucleocytoplasmic shuttling and regulates human embryonic stem-cell self-renewal. Nat Cell Biol 2008;10:837–848. 18568018 10.1038/ncb1748

[CIT0052] Wagle MV , Marchingo JM, Howitt J et al. The ubiquitin ligase adaptor NDFIP1 selectively enforces a CD8(+) T cell tolerance checkpoint to high-dose antigen. Cell Rep 2018;24:577–584. 30021156 10.1016/j.celrep.2018.06.060 PMC6112980

[CIT0053] Xie H , Wu L, Deng Z et al. Emerging roles of YAP/TAZ in lung physiology and diseases. Life Sci 2018;214:176–183. 30385178 10.1016/j.lfs.2018.10.062

[CIT0054] Yang S , Dong Q, Yao M et al. Establishment of an experimental human lung adenocarcinoma cell line SPC-A-1BM with high bone metastases potency by (99m)Tc-MDP bone scintigraphy. Nucl Med Biol 2009;36:313–321. 19324277 10.1016/j.nucmedbio.2008.12.007

[CIT0055] Yu Y , Song Z, Yang S et al. Everolimus and zoledronic acid—a potential synergistic treatment for lung adenocarcinoma bone metastasis. Acta Biochim Biophys Sin (Shanghai) 2014;46:792–801. 25098623 10.1093/abbs/gmu069

[CIT0056] Zanconato F , Cordenonsi M, Piccolo S. YAP/TAZ at the roots of cancer. Cancer Cell 2016;29:783–803. 27300434 10.1016/j.ccell.2016.05.005 PMC6186419

[CIT0057] Zhang Y , Li D, Jiang Q et al. Novel ADAM-17 inhibitor ZLDI-8 enhances the in vitro and in vivo chemotherapeutic effects of Sorafenib on hepatocellular carcinoma cells. Cell Death Dis 2018;9:743. 29970890 10.1038/s41419-018-0804-6 PMC6030059

[CIT0058] Zhang L , Zhang S, Yao J et al. Microenvironment-induced PTEN loss by exosomal microRNA primes brain metastasis outgrowth. Nature 2015;527:100–104. 26479035 10.1038/nature15376 PMC4819404

[CIT0059] Zhang YY , Zhang CB, Zhao Q et al. The miR-873/NDFIP1 axis promotes hepatocellular carcinoma growth and metastasis through the AKT/mTOR-mediated Warburg effect. Am J Cancer Res. 2019;9:927. 31218102 PMC6556606

