# Supplementary material for: NDFIP1 limits cellular TAZ accumulation via exosomal sorting to inhibit NSCLC proliferation
Source: Protein Cell. 2022 Sep 21;14(2):123–36. doi: 10.1093/procel/pwac017 (PMC10019574; doi:10.1093/procel/pwac017)
Supplement: pwac017_suppl_Supplementary_Material [file pwac017_suppl_supplementary_material.docx]

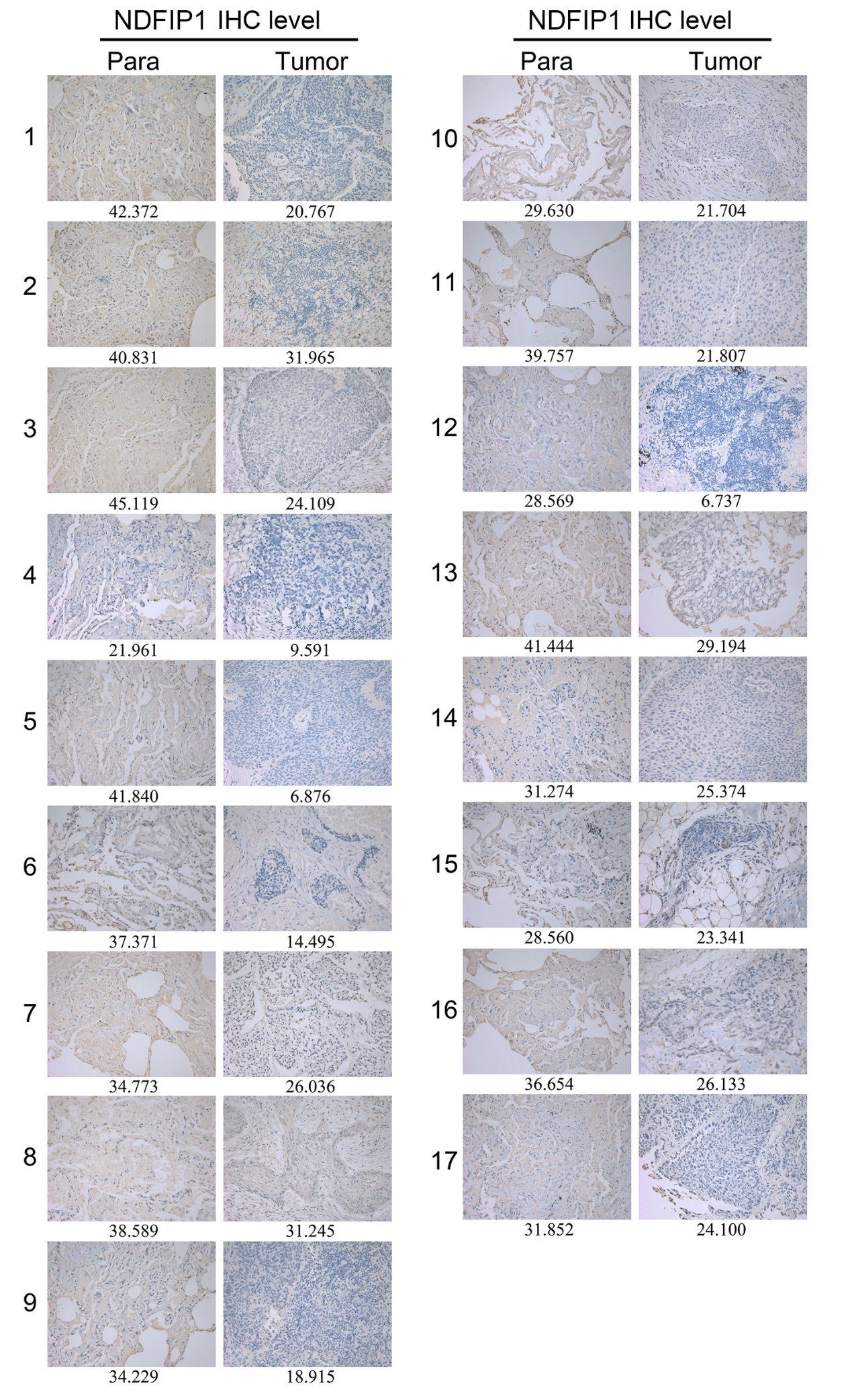


**Supplementary Figure 1**. The protein level of NDFIP1 in 17 NSCLC and paired paratumor samples. The quantification of IHC staining intensity of NDFIP1 is shown below each graph.


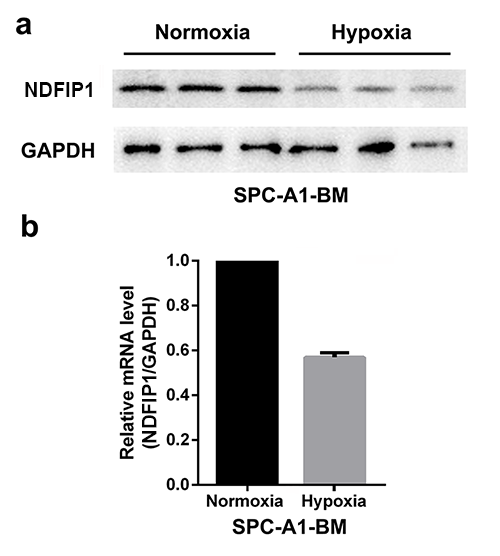


**Supplementary Figure 2**. Hypoxia induces the lower protein and mRNA level of NDFIP1. (a), NDFIP1 protein level in SPC-A1-BM cells in the normoxia and hypoxia conditions. (b), the NDFIP1 mRNA levels in SPC-A1-BM cells in the normoxia and hypoxia conditions. Bars represent the mean ± SD (*n* = 3).


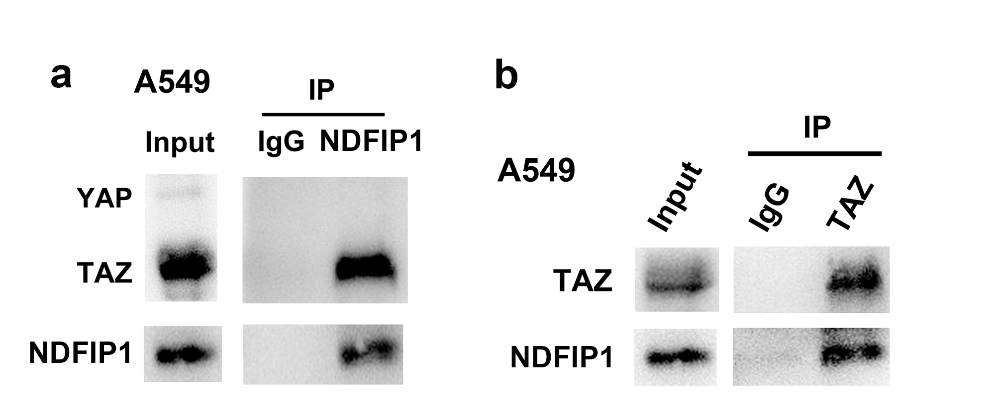


**Supplementary Figure 3**. NDFIP1 interacts with TAZ in A549 cells. (a), the binding of NDFIP1 to TAZ. (b), the converse binding of TAZ to NDFIP1.


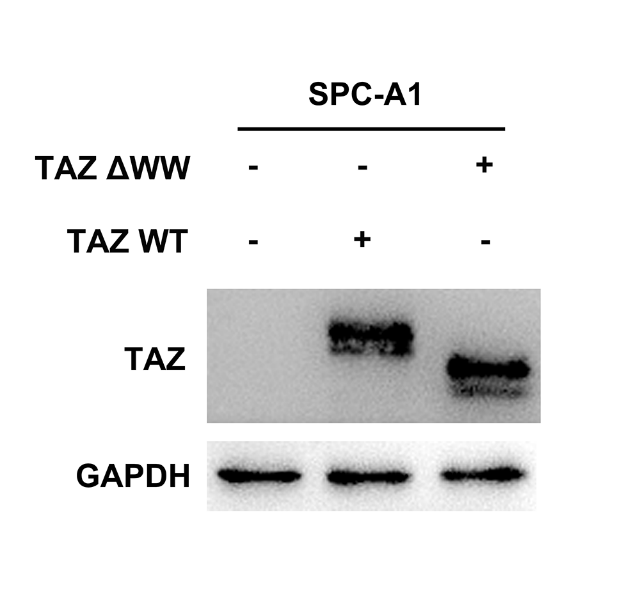


**Supplementary Figure 4**. SPC-A1 cells were transfected with TAZ WT or TAZ ΔWW plasmid (deletion of WW-domain).


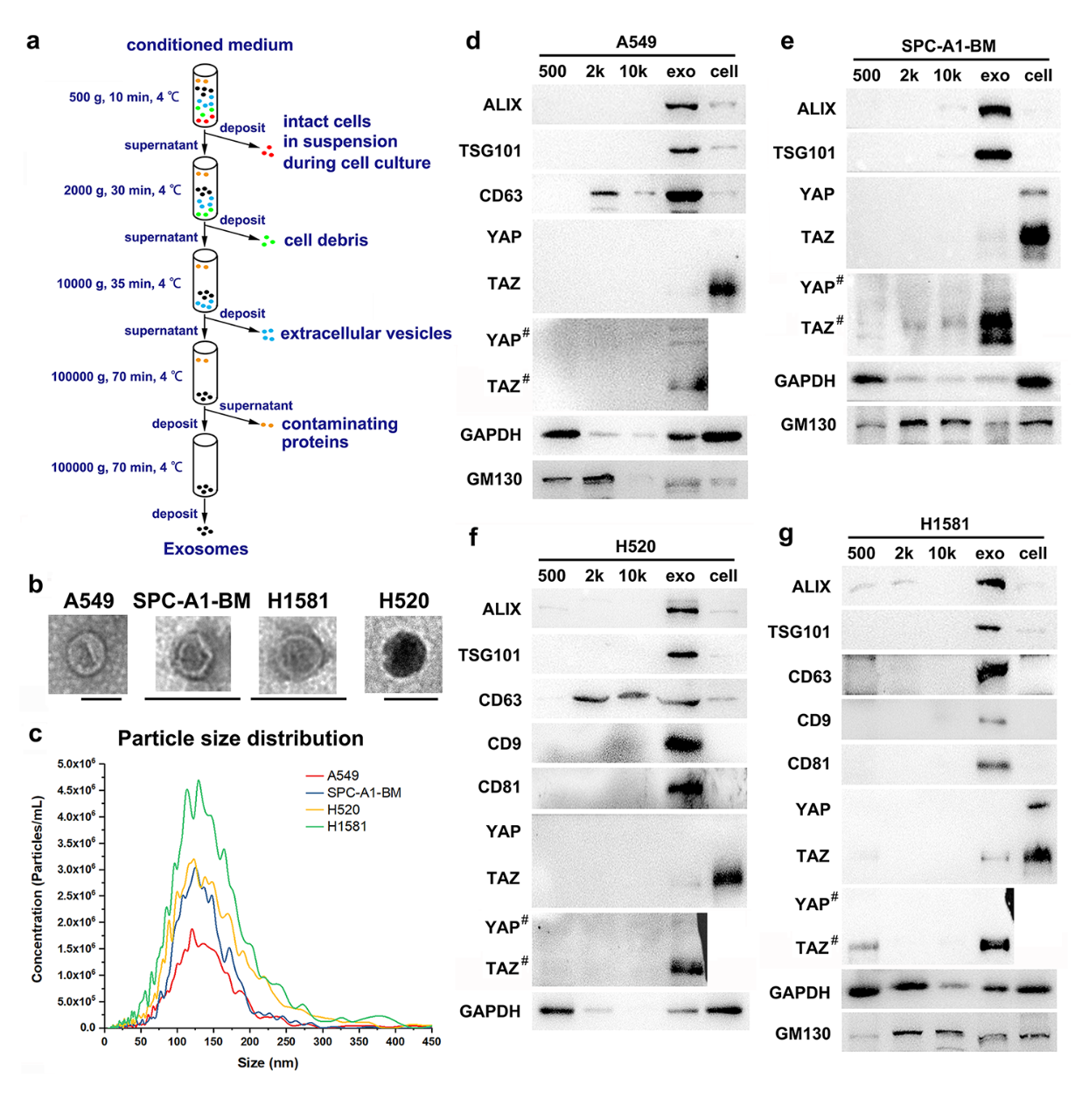


**Supplementary Figure 5**. TAZ exists in exosomes from lung cancer cells. (a), the flowchart of exosome isolation by ultracentrifugation method. (b), TEM image of exosomes from A549, SPC-A1-BM, H1581, H520 cells. Scale bars: 100 nm. (c), the particle size distribution of exosomes from A549, SPC-A1-BM, H1581 and H520 cells. (d-g), the protein levels of YAP, TAZ, exosome-specific proteins, ALIX, TSG101, CD63, CD9, CD81, a cell internal reference, GAPDH and a Golgi marker, GM130 in different components of A549, SPC-A1-BM, H1581 and H520, including the pellets after centrifugation at 500 g, 2000 g, 10000 g as well as exosomes and whole cell. ^#^ represents longer exposure.


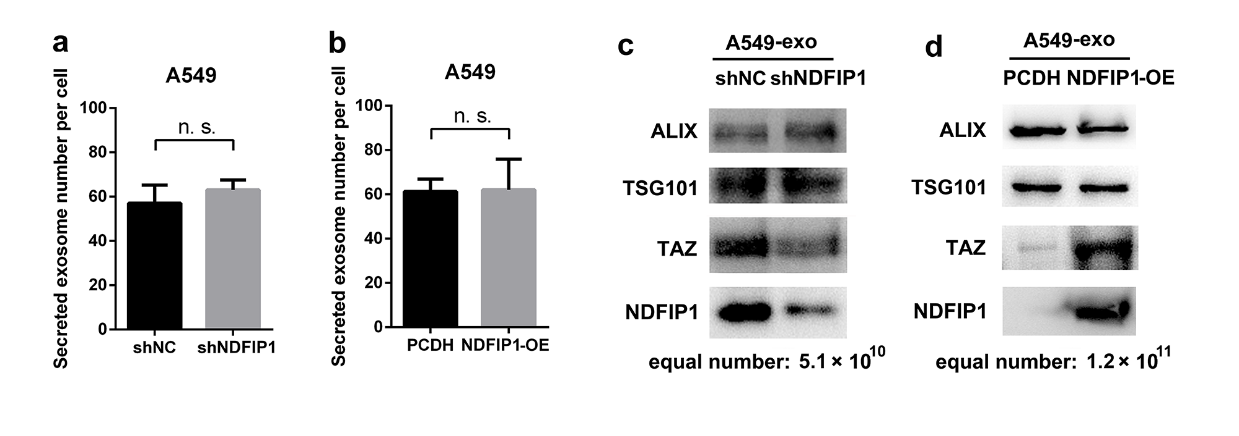


**Supplementary Figure 6**. NDFIP1 determines exosomal sorting of TAZ, but not the secretion of exosomes. (a-b), the average number of secreted exosomes per cell of (a) the control (shNC) and NDFIP1 knockdown (shNDFIP1) and (b) the control (PCDH) and NDFIP1 overexpression (NDFIP1-OE) A549 cells. Bars represent the mean ± SD (*n* = 3). (a) n. s. *P* = 0.2759. (b) n. s. *P* = 0.9476. (c-d), the TAZ and NDFIP1 protein levels in equal number of exosomes from the (c) control (shNC) and NDFIP1 knockdown (shNDFIP1) A549 cells, and (d) from the control (PCDH) and NDFIP1 overexpression (NDFIP1-OE) A549 cells.


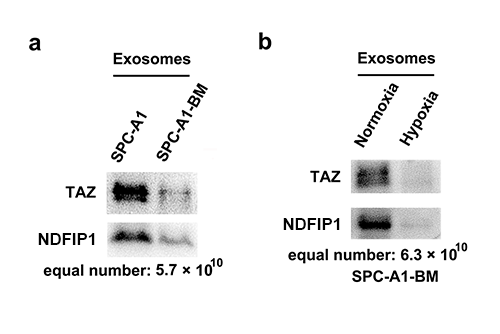


**Supplementary Figure 7**. Tumor cells with lower NDFIP1 secretes lower TAZ in equal exosomes. (a), the TAZ and NDFIP1 protein levels in equal number of exosomes: 5.7 × 10^10^ of exosomes from SPC-A1 and SPC-A1-BM cells. (b) the TAZ and NDFIP1 protein levels in equal number of exosomes: 6.3 × 10^10^ of exosomes from SPC-A1-BM cells in the normoxia and hypoxia conditions.


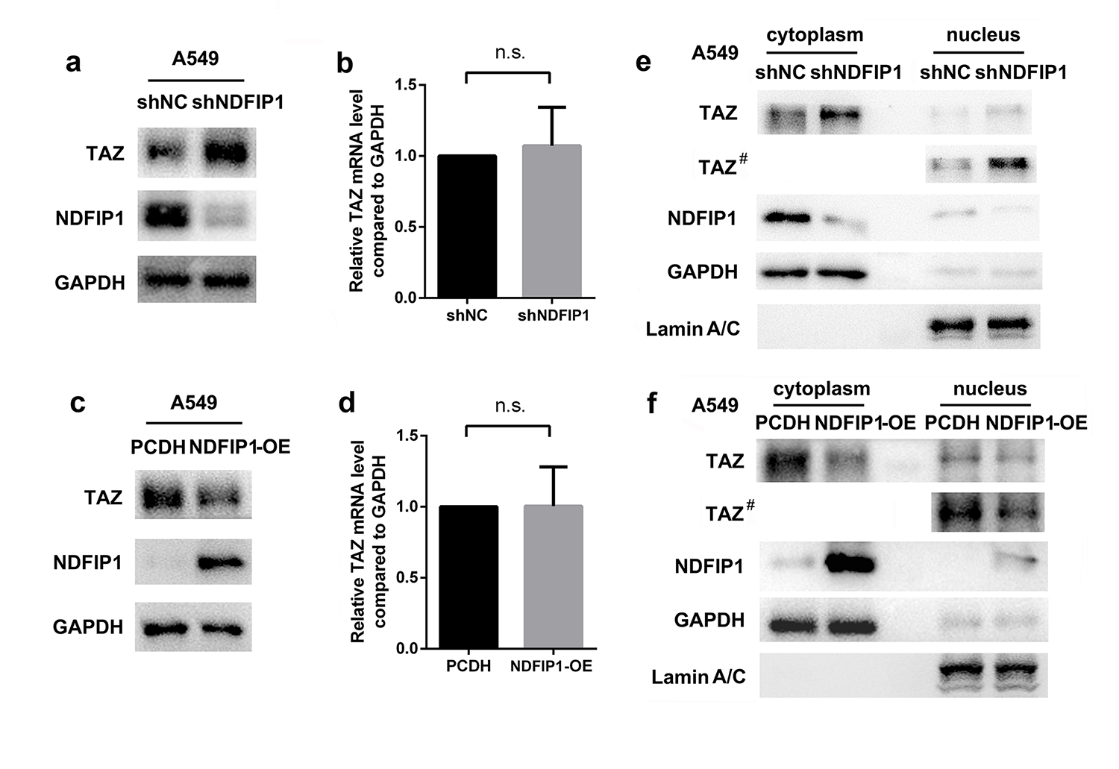


**Supplementary Figure 8**. NDFIP1 negatively regulates TAZ in A549 cells without significant changes in TAZ mRNA level. (a, c), protein levels of TAZ and NDFIP1 in (a) control (shNC) and NDFIP1 knockdown (shNDFIP1) A549 cells, (c) control (PCDH) and NDFIP1 overexpression (NDFIP1-OE) A549 cells. (b, d), relative mRNA levels of TAZ in these four groups. Bars represent the mean ± SD (*n* = 3). (e-f), protein levels of TAZ and NDFIP1 in the cytoplasm and the nucleus fraction in these four groups. ^#^ represents longer exposure.


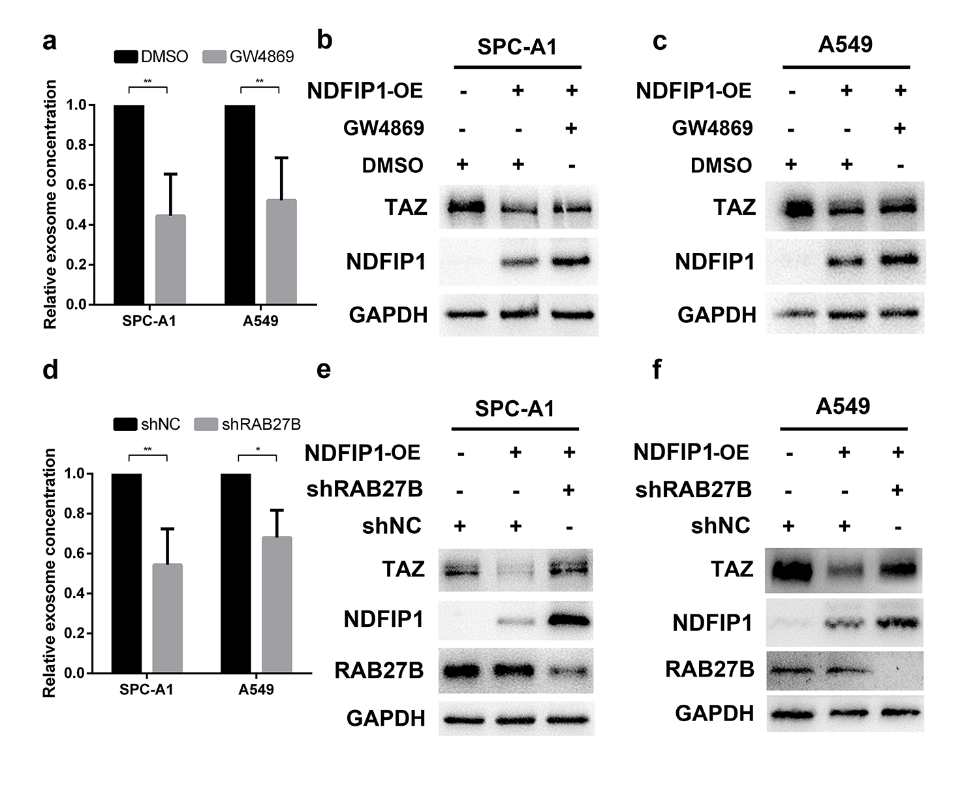


**Supplementary Figure 9**. The decrease of TAZ induced by NDFIP1 overexpression can be eliminated by inhibiting the secretion of exosomes. (a), the relative number of exosomes secreted by SPC-A1 and A549 cells with or without GW4869. ***P* < 0.01. (b), the TAZ protein level in PCDH and NDFIP1-OE SPC-A1 cells with or without GW4869. (c), the TAZ protein level in PCDH and NDFIP1-OE A549 cells with or without GW4869. (d), the relative number of exosomes secreted by SPC-A1 and A549 cells with or without RAB27B knockdown. **P* < 0.05. ***P* < 0.01. (e), the TAZ protein level in PCDH and NDFIP1-OE SPC-A1 cells with or without RAB27B knockdown. (f), the TAZ protein level in PCDH and NDFIP1-OE A549 cells with or without RAB27B knockdown.


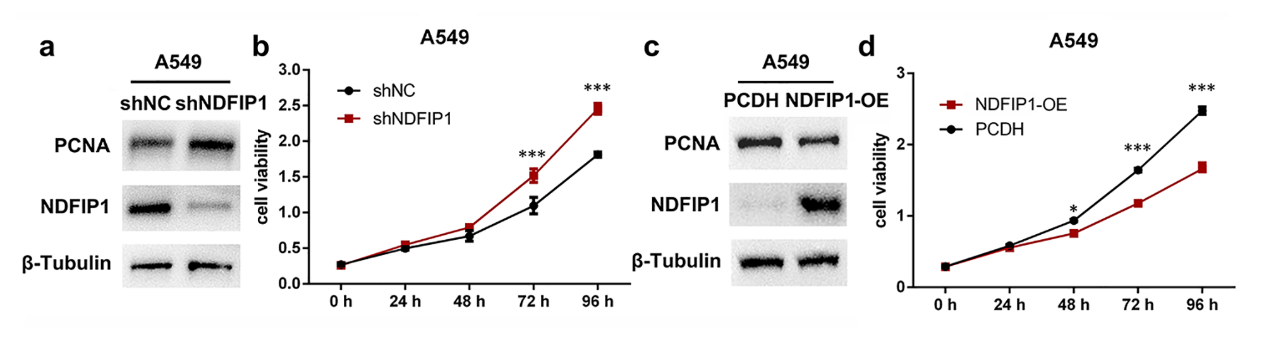


**Supplementary Figure 10**. NDFIP1 inhibits cell proliferation in A549 cells. (a, c), protein levels of a proliferation marker, PCNA in (a) control (shNC) and NDFIP1 knockout (shNDFIP1) A549 cells, (c) control (PCDH) and NDFIP1 overexpression (NDFIP1-OE) A549 cells. (b, d), cell viability assay of these four groups. Bars represent the mean ± SD (*n* = 3). **P* < 0.05. ****P* < 0.001.


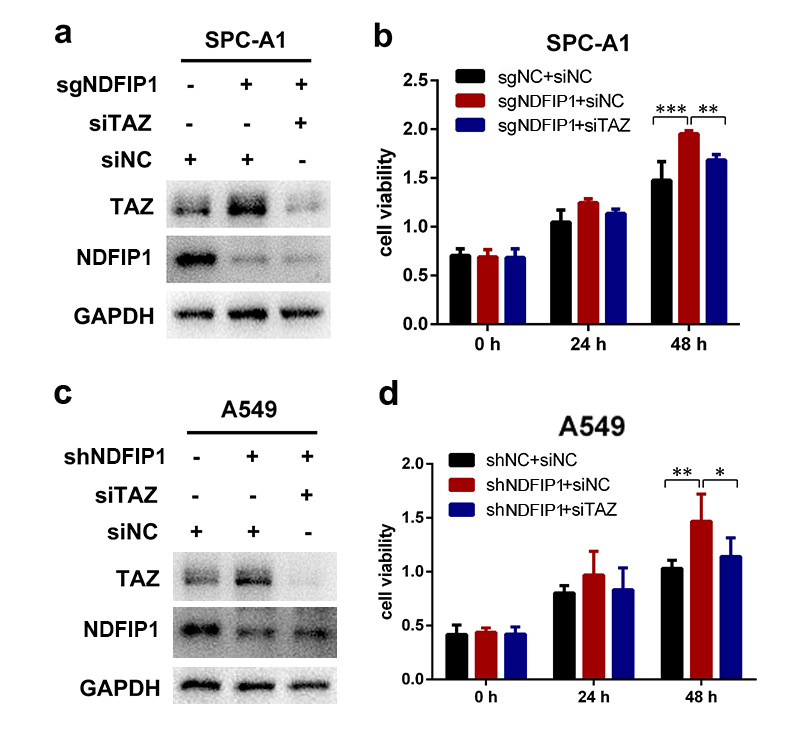


**Supplementary Figure 11**. Interfering TAZ inhibits NDFIP1 downregulation induced cell proliferation. (a), the protein levels of TAZ and NDFIP1 in control (sgNC) and NDFIP1 knockout (sgNDFIP1) SPC-A1 cells with the transfection of siNC or siTAZ. (d), cell viability assay of these three groups in SPC-A1 cells. Bars represent the mean ± SD (*n* = 3). ***P* < 0.01. ****P* < 0.001. (c), the protein levels of TAZ and NDFIP1 in control (shNC) and NDFIP1 knockout (shNDFIP1) A549 cells with the transfection of siNC or siTAZ. (d), cell viability assay of these three groups in A549 cells. Bars represent the mean ± SD (*n* = 3). **P* < 0.05. ***P* < 0.01.


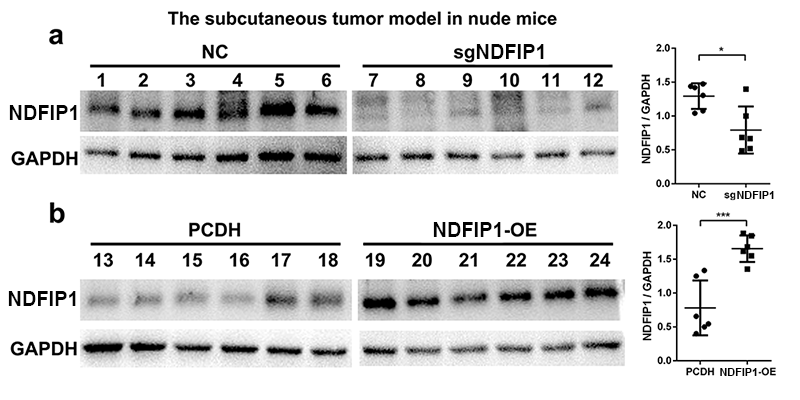


**Supplementary Figure 12**. The NDFIP1 protein level in tumors harvested from the mouse xenograft model. (a-b), the NDFIP1 protein level in subcutaneous tumors from mice receiving (a) control (sgNC) and NDFIP1 knockout (sgNDFIP1) SPC-A1 cells, (b) control (PCDH) and NDFIP1 overexpression (NDFIP1-OE) SPC-A1 cells. The relative quantification of NDFIP1 is plotted on the right. Bars represent the mean ± SD (*n* = 6). **P* < 0.05. ****P* < 0.001.


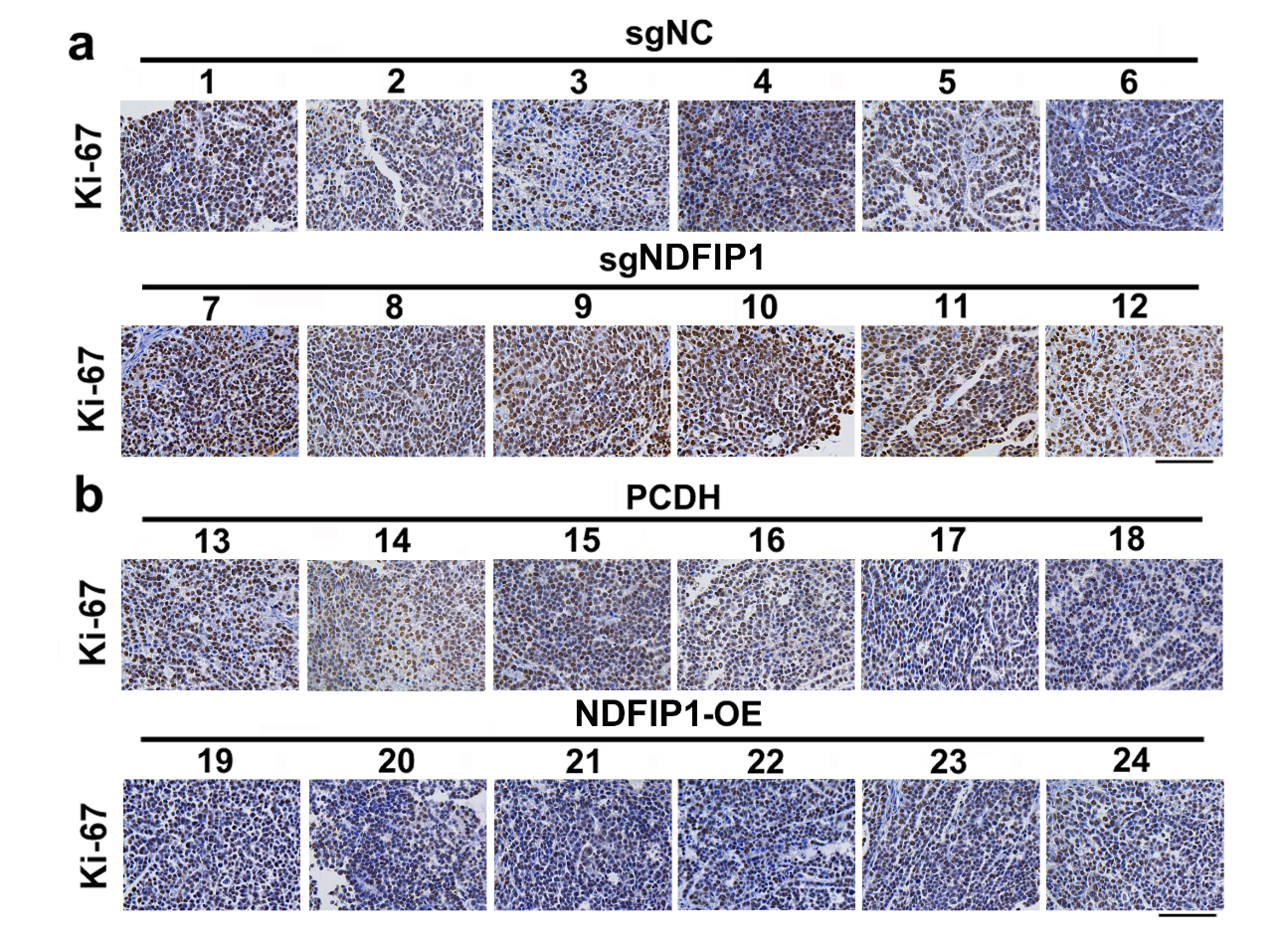


**Supplementary Figure 13**. The Ki-67 protein level in tumors harvested from the mouse xenograft model. (a-b), the IHC images of tumors from subcutaneous mouse xenograft model of (a) control (sgNC) and NDFIP1 knockout (sgNDFIP1) SPC-A1 cells, (b) control (PCDH) and NDFIP1 overexpression (NDFIP1-OE) SPC-A1 cells. Scale bars: 100 μm.


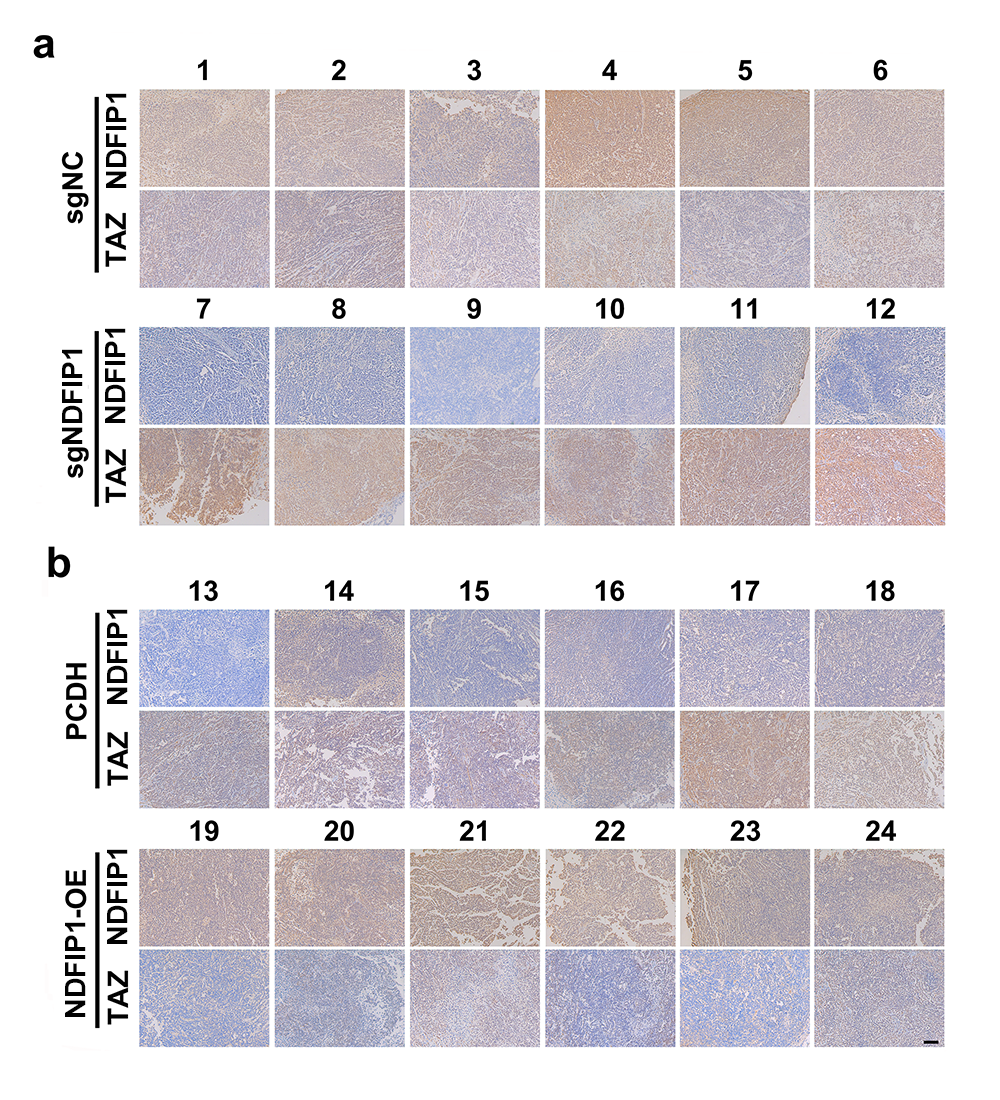


**Supplementary Figure 14**. IHC staining of cellular NDFIP1 and TAZ in subcutaneous tumors. (a-b), IHC staining of NDFIP1 and TAZ in subcutaneous tumors from mice receiving (a) control (sgNC) and NDFIP1 knockout (sgNDFIP1) SPC-A1 cells, (b) control (PCDH) and NDFIP1 overexpression (NDFIP1-OE) SPC-A1 cells. Scale bars: 200 μm.


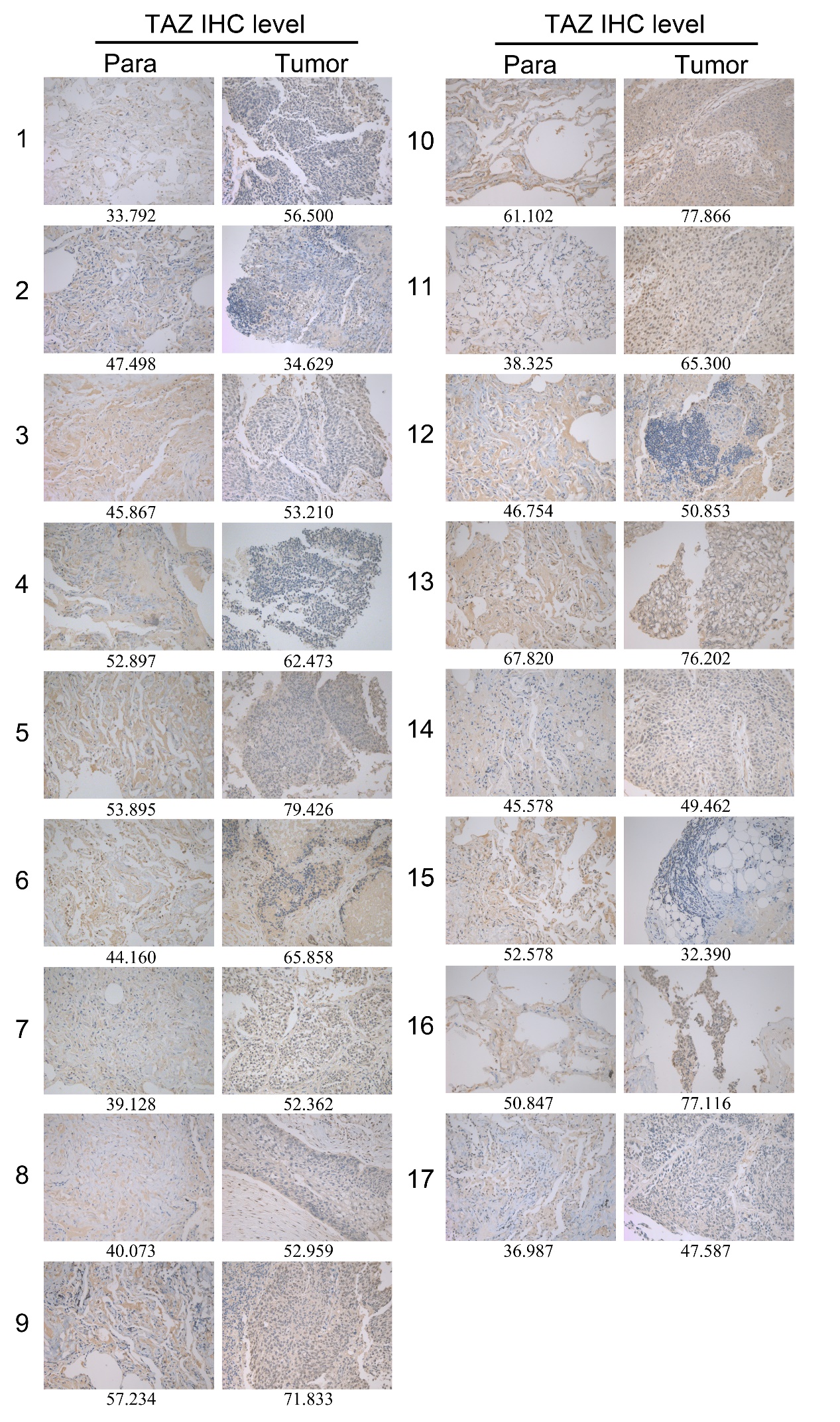


**Supplementary Figure 15**. The protein level of TAZ in 17 NSCLC and paired paratumor samples. The quantification of IHC staining intensity of TAZ is shown below each graph.


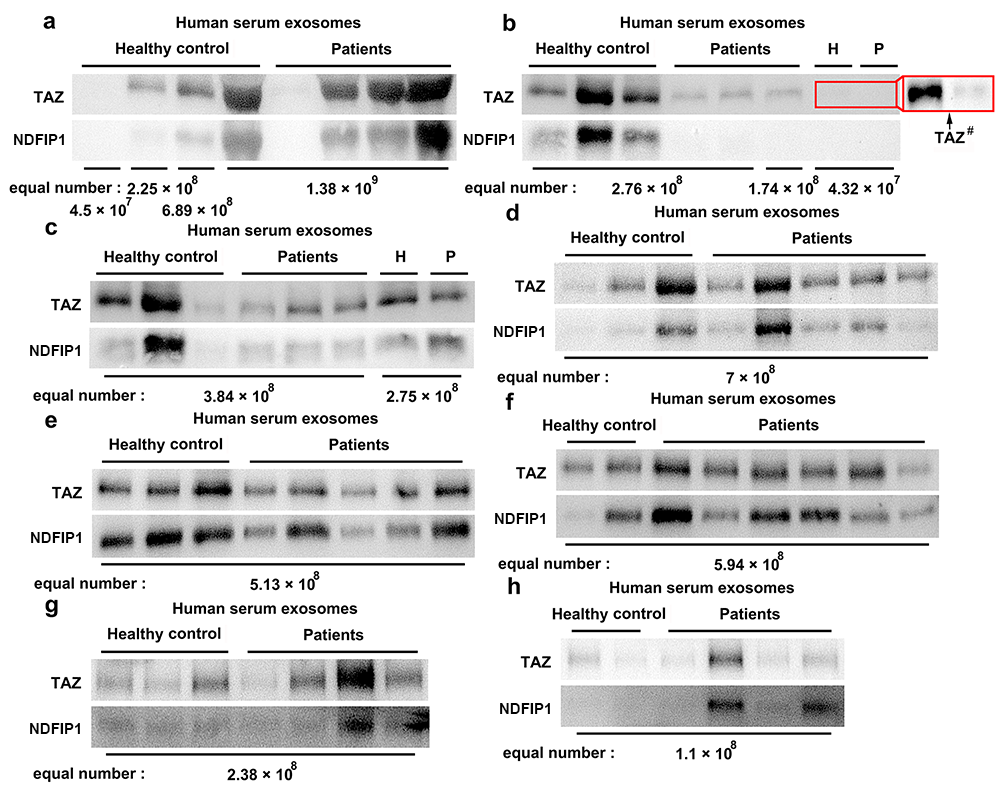


**Supplementary Figure 16**. The TAZ and NDFIP1 protein level in human serum exosomes. (a-h), the TAZ and NDFIP1 protein levels in equal number of serum exosomes from the healthy control (H) and NSCLC patients (P). H=Healthy control, P=Patients. ^#^ represents longer exposure.
